# Variation in adult and pup wolf diets at natal den sites is influenced by forest composition and configuration

**DOI:** 10.1002/ece3.9648

**Published:** 2023-01-11

**Authors:** Gretchen H. Roffler, Kristine L. Pilgrim, Katherine E. Zarn, Michael K. Schwartz, Taal Levi

**Affiliations:** ^1^ Division of Wildlife Conservation Alaska Department of Fish and Game Douglas Alaska USA; ^2^ National Genomics Center for Wildlife and Fish Conservation Rocky Mountain Research Station, USDA Forest Service Missoula Montana USA; ^3^ Department of Fisheries and Wildlife Oregon State University Corvallis Oregon USA; ^4^ Present address: National Technology and Development Program USDA Forest Service Flagstaff Arizona USA

**Keywords:** *Canis lupus*, dens, diet, DNA metabarcoding, noninvasive sampling, *Odocoileus hemionus sitkensis*

## Abstract

Although wolves are wide‐ranging generalist carnivores throughout their life cycle, during the pup‐rearing season wolf activity is focused on natal den sites where pup survival depends upon pack members provisioning food. Because prey availability is influenced by habitat quality within the home range, we investigated the relative importance of prey species for adults and pups and further examined the relationship between habitat characteristics, wolf diet, and litter size on Prince of Wales Island (POW) in Southeast Alaska. During 2012–2020, we detected 13 active den sites within the home ranges of nine wolf packs. We estimated minimum pup counts using motion‐detecting cameras and individual genotypes from noninvasive samples (hair: *n* = 322; scat: *n* = 227) and quantified wolf diet composition using fecal DNA metabarcoding (*n* = 538). We assessed habitat composition, configuration, and connectivity within denning and annual home ranges estimated using wolf GPS‐collar data. Contrary to expectations, wolves had a more constricted diet during denning season (April 15–July 31), and within this season pups had a narrower dietary niche (species richness [*S*] = 4) focused more on deer (relative frequency of occurrence [O/I] = 0.924) than adults (*S* = 15; deer O/I = 0.591). Litter size had a positive relationship with the relative frequency of deer in a wolf pack's diet. Wolf consumption of deer was positively associated with the proportion of young‐growth forest (≤25 years old) within denning and annual home ranges. High levels of vegetation patch interspersion, and the density of closed logging roads were also important predictors, suggesting these habitat qualities were influential for increasing the availability of deer to wolves. Our results contrast with previous research indicating wolf pup diets included more alternate prey (i.e., beaver) than adults and emphasize the importance of deer to wolf viability on POW, especially during denning season.

## Graphical abstract

Despite demonstrated dietary plasticity throughout their life cycle, wolves had a narrow dietary niche during the denning season focused on Sitka black‐tailed deer (*Odocoileus hemionus sitkensis*), and pups nearly exclusively consumed deer during their first 2 months of life. Wolf consumption of deer was influenced by the availability of early successional forest, closed logging road density, and forest patchiness, suggesting these habitat qualities were influential for increasing the availability of deer to wolves. The contribution of deer in wolf diets had a positive effect on litter size providing a link to wolf fitness.

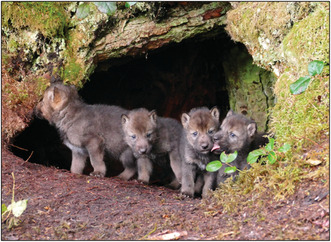

## 1 INTRODUCTION

Wolves are renowned for their behavioral and dietary plasticity (Peterson & Ciucci, 2003), allowing them to acquire resources in a variety of habitats (Mech & Peterson, 2003). However, wolf activity patterns and space use become focused on natal sites during denning season as pups have reduced mobility (Mech & Boitani, 2003; Packard, 2003). Pups are dependent on milk during the first 5 weeks of life after which time they begin to develop teeth and the ability to digest food regurgitated and carried to them by adults (Packard, 2003). Because adult wolves, and especially breeding wolves (Mech & Boitani, 2003; Packard, 2003), need to make frequent trips to the den site to provision pups, their movements become constricted and their core use areas around den sites are typically smaller than during the rest of the year (Roffler & Gregovich, 2018). Non‐breeding wolves also play an important role in both attending and provisioning the pups especially before weaning due to the limited ability of the breeding female to leave the den for extended periods (Packard, 2003; Ruprecht et al., 2012). The capacity of the habitat surrounding the den site to support prey species is therefore important for providing foraging opportunities for wolves and to promote efficient prey acquisition (Harrington et al., 1983). Variation in diet among wolf packs even in adjacent home ranges is likely influenced by differences in prey availability and abundance in each pack (Gable et al., 2017; Lodberg‐Holm et al., 2021).

**FIGURE 1:**
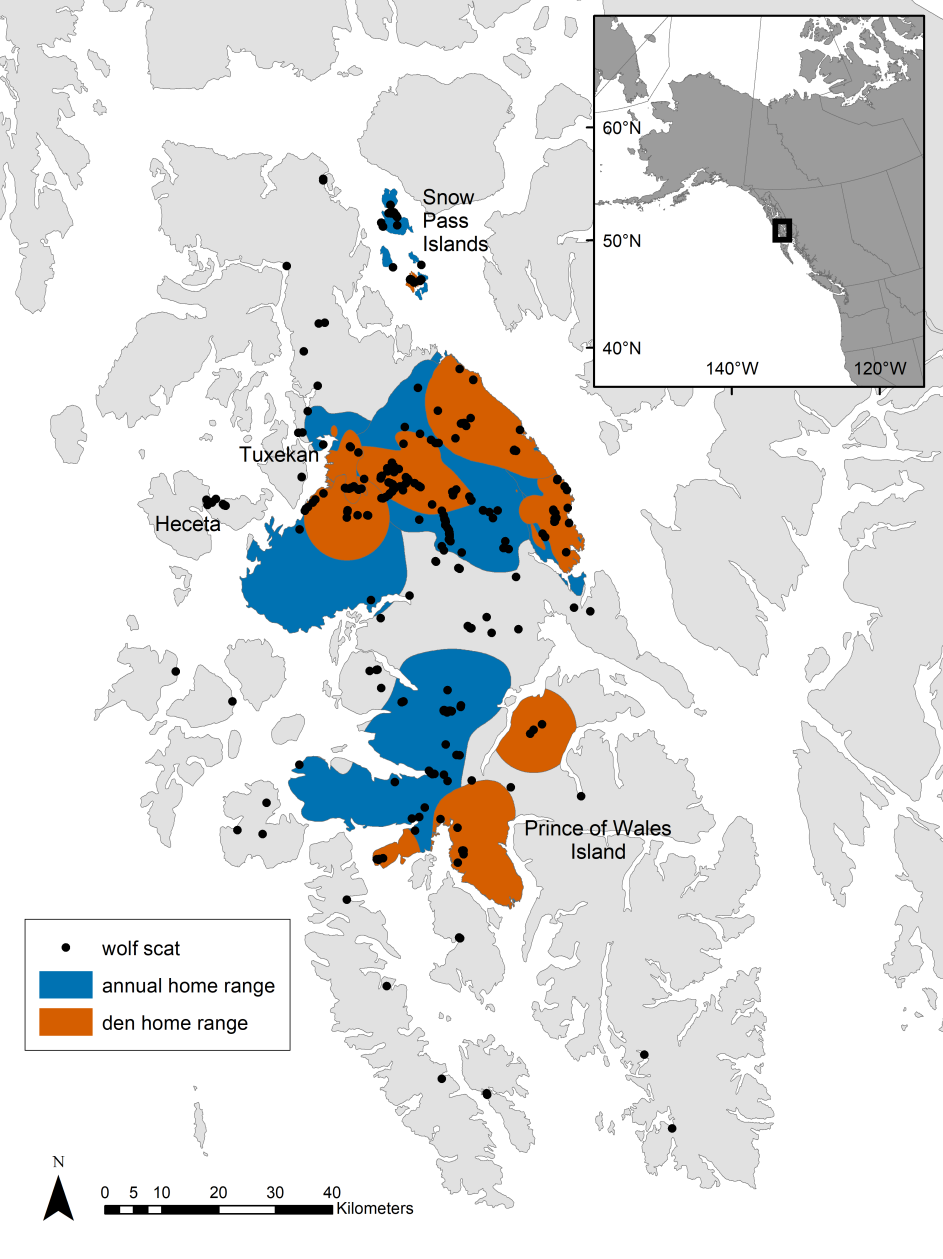
Wolf scat locations and denning and annual home ranges. Prince of Wales Island, Alaska 2014–2020

Variation in reproductive and survival rates are factors that contribute to population viability, a relevant topic in status assessments of the Alexander Archipelago wolf (*Canis lupus ligoni*) in Southeast Alaska. This subspecies of wolf has been the focus of conservation concerns since the 1990s, resulting in three petitions for listing as threatened under the Endangered Species Act, the most recent in 2020 (USFWS, 2020). Management and conservation attention has focused on Prince of Wales Island (POW; Figure 1) due to the concentration of extensive old‐growth logging and habitat fragmentation expected to be detrimental to Sitka black‐tailed deer (*Odocoileus hemionus sitkensis*), the primary prey of wolves. If deer populations decline following large‐scale reductions in habitat capability as logged forests transition into closed‐canopy second‐growth forest (Alaback, 1982; Farmer & Kirchhoff, 2007), it is predicted that wolf population viability would also decrease (Gilbert et al., 2022; Person, 2001). Previous work has demonstrated that although deer are the primary prey of wolves on POW, they also consume a broad variety of prey items and may adjust their diets based on season or prey availability in forests under different management regimes (Kohira & Rexstad, 1997; Massey et al., 2021; Roffler et al., 2021; Szepanski et al., 1999). However, wolf summer diets are relatively understudied (Peterson & Ciucci, 2003) especially during denning season, and little is known about the diets of wolf pups (Bryan et al., 2006; Paquet & Carbyn, 2003). Previous research suggested that wolf pups have a broader dietary niche than adults, indicating selective provisioning of pups (Bryan et al., 2006; Sidorovich et al., 2017). Pup survival is variable during early denning season (Fuller et al., 2003; Harrington et al., 1983), and may be influenced by a quantity and quality of food brought to them (Benson et al., 2013; Van Ballenberghe & Mech, 1975). Therefore, understanding the relative importance of prey species for adults and pups during the denning season and the relationship between wolf diet and habitat characteristics may provide insight into the relevant conditions for reproduction and pup‐rearing. A question remaining to be answered is how dietary plasticity affects key fitness characteristics such as wolf reproduction and survival when the relative contribution of the primary ungulate prey is reduced.

To gain insights into the diets of wolf pups and adults during denning season, we investigated wolves on POW during 2014–2020 and conducted intensive data collections at active den sites during 2015–2018. We quantified wolf diet composition using DNA metabarcoding of wolf scats. We first compared summer and winter diets including scats collected within home ranges of wolf packs. We then compared wolf diets restricted both temporally to the denning season (April 15–July 31) and spatially to the den site (within 100 m) to wolf diets during the rest of the year and throughout the entire annual home range. We gained information about variation in litter size using noninvasive samples to obtain individual genotypes and images from motion‐detecting cameras. To further examine the relationship between habitat characteristics and wolf diet, we assessed habitat composition, configuration, and connectivity within wolf denning and annual home ranges. We expected wolf diets would be more diverse during the summer (Newsome et al., 2016; Peterson & Ciucci, 2003) and that pup diets would be more diverse than adults based on previous research. We further expected wolf packs with home ranges containing more favorable deer habitat (low‐volume old‐growth forests and young successional clearcuts) to have a higher relative contribution of deer in their diets.

## 2 STUDY AREA

We studied wolves on POW and the surrounding islands in the Southeast Alaska Archipelago (Figure 1). This large island (6670 km^2^) is characterized by temperate rainforests with high annual precipitation (130–400 cm). Sitka spruce (*Picea sitchensis*) and western hemlock (*Tsuga heterophylla*) are the dominant forest species and occur in a mosaic of old‐growth stands interspersed with even‐aged forest at varying successional stages resulting from clearcut logging. POW has experienced the highest rates of industrial logging in Southeast Alaska, with reductions of contiguous high‐volume forests by 94% during 1954–2004 (Albert & Schoen, 2013). An extensive network of logging roads (approximately 4800 km) transects POW with densities highest in the northern portion of the island (0–4.44 km/km^2^; Roffler et al., 2018). Wolf density on POW and the surrounding islands ranged from 10 to 44 wolves/1000 km^2^ during 2013–2020 (Dorendorf, 2021; Roffler et al., 2019). Along with Sitka black‐tailed deer (*Odocoileus hemionus*), the terrestrial mammals contributing to wolf diets on POW include American black bear (*Ursus americanus*), North American beaver (*Castor canadensis*), American marten (*Martes americana*), river otter (*Lontra canadensis*), and small mammals (*Microtus* spp.), in addition to salmonids (*Oncorhynchus* spp.), and a variety of bird species (Kohira & Rexstad, 1997; Massey et al., 2021; Roffler et al., 2021).

## 3 METHODS

### 3.1 Den monitoring

During 2012–2020, we monitored wolf dens on POW and the surrounding islands in the complex including the Snow Pass Islands, Heceta, and Tuxekan (Figure 1) that had been identified from locations of GPS‐collared wolves or from previously recorded den sites (Person & Russell, 2009; Roffler & Gregovich, 2018). We investigated 4–22 den sites annually and identified between 1 and 5 active dens (i.e., reproducing wolves and pups present) each year. We installed motion‐detecting cameras (Reconyx HC600, Reconyx, Inc. or Moultrie M990i, Moultrie Products) at the den sites to record activity. During 2015–2018, we collected wolf hair and scat at active dens to obtain DNA for individual identification. We intensively searched the area within a 100 m radius of the active den site for scat and hair samples, assuming that pups would not travel outside of this zone during the early denning season. We estimated minimum pup and adult (>1 year old) counts at each active den (i.e., the highest number of wolves observed) using a combination of images from cameras, individual genotypes from noninvasive samples, and ground‐based observations. We also used the same scats to identify diet items. Once an active den was identified, we conducted 2–3 collection sessions between mid‐May and mid‐July to ensure sufficient hair and scat samples could be obtained. On POW, pups are born in late April through early May (mean den entry date May 2; Roffler & Gregovich, 2018), thus we ensured the timing of our collection trips was after pups would have reached 2–3 weeks of age. We collected pup scats from early June through mid‐July when pups would have transitioned from milk to solid food in order to quantify the contribution of prey species to their diets. We distinguished between adult (>2.5 cm) and pup scat (<2.5 cm) due to distinct differences in age class size during denning season (Weaver & Fritts, 1979). Scats were placed in paper bags and stored in plastic bins or resealable plastic bags with silica gel for desiccation, and hair samples were stored in labelled coin envelopes and stored at room temperature, until all samples could be shipped to the National Genomics Center for Wildlife and Fish Conservation, Missoula, MT, USA.

### 3.2 Genotyping

DNA extractions, genotyping, and sex identification of wolf samples were conducted at the National Genomics Center for Wildlife and Fish Conservation and previously described in Roffler et al. (2019). Briefly, DNA was extracted from samples using standard protocols for tissues (DNeasy Blood & Tissue kit; Qiagen) with the following modifications: overnight incubation in buffer ATL and Proteinase K on a rocker or in a rotating oven at 56°C, a 70°C incubation for 10 min after adding buffer AL, and a final elution using 100 μl buffer AE warmed to 70°C. When DNA from samples failed to amplify, we re‐extracted the DNA to increase the chance of genotyping success for those individuals. We analyzed hair and scat DNA extractions for individual identification using a panel of 15 variable microsatellite loci: cph5 (Fredholm & Wintero, 1995); fh2001, fh2010, fh2054, fh2079, fh2088, fh2096, fh2137, fh2140, fh2161, fh2548 (Francisco et al., 1996), Pez17, (Duchamp et al., 2012); c20.253 (Ostrander et al., 1993), VWF (Shibuya et al., 1994), AHT130 (Holmes et al., 1995). Samples that amplified with three or more alleles at a single locus (indicating a mixture of one or more individuals) or that failed to be genotyped at seven or more loci were discarded. We used vertebrate primers to amplify a 360 bp 16S rRNA region of the mitochondrial genome, performed Sanger sequencing, and used NCBI BLAST to distinguish canid samples from non‐target species (e.g., bears). The sex of individual wolves was identified using the canid SRY marker (Wictum et al., 2013).

After genotyping, we used DROPOUT v. 2.3 (McKelvey & Schwartz, 2005) and AlleleMatch (Galpern et al., 2012) in R (v. 4.0.3, R Core Team) to highlight individuals with incomplete loci matches and to identify allele scoring or data entry errors. We used MicroChecker v. 2.2.3 (Van Oosterhout et al., 2004) to check for homozygote excess due to null alleles and identify possible scoring errors. We used GenAlEx v. 6.503 (Peakall & Smouse, 2012) to evaluate allele frequencies, verify low‐frequency alleles, and to confirm samples collected were from wolves and not domestic dogs. This was done by generating a principal coordinates analysis (PCoA) graph showing known dogs, known wolves, and the hair and scat samples collected at den sites. Reference genotypes for the PCoA were from captured and harvested Southeast Alaska wolves (*n* = 159) and North American domestic dogs (*n* = 88). We also performed assignment tests using GeneClass2 (Piry et al., 2004) on all hair and scat samples and the same reference samples to verify that all hair and scat samples collected at den sites were deposited by wolves and not dogs. All den check hair and scat samples assigned to the wolf population. The 15 loci gave a cumulative probability of individual identity and probability of identity giving siblings as 2.17 × 10^−10^ and 6.074 × 10^−5^, respectively.

### 3.3 Wolf diet composition

In addition to collecting wolf scats at den sites, we also collected scats throughout the year along wolf travel routes (e.g., game trails, river corridors, beaches, etc.) and on secondary roads while conducting other wolf monitoring field work during 2014–2021. We estimated the age class (fresh [<1 week], medium‐aged [>1 week to <3 months], old [>3 months]) of scat based on appearance and time since last site visit (Ciucci et al., 1996) and exposure time considering that scats decompose rapidly in rainforest environments (Darimont et al., 2004). Collected scats were stored in resealable plastic bags, labelled with location, date, and perceived age of the scat prior to analysis, and frozen (−20°C). Frozen scats and scats collected at den sites were shipped to Oregon State University for sample preparation and analysis.

We used metabarcoding of amplified target DNA sequences in wolf scats to identify wolf prey items adhering to previously described procedures (Massey et al., 2021; Roffler et al., 2021) summarized below. We pooled three subsamples collected from the middle interior section of each scat (total quantity = 200 mg) and used a slightly modified extraction protocol (Roffler et al., 2021) from the Qiagen DNeasy Blood and Tissue kit (Qiagen). We included a blank control in each extraction batch to identify possible cross contamination. We identified vertebrate species consumed by wolves used slightly modified primers (Riaz et al., 2011) to amplify a ~100 bp region of the mitochondrial 12S region. Forward and reverse primers were labelled with identical indexes, and three PCR replicates were independently sequenced as previously described (Roffler et al., 2021). We quantified and normalized DNA concentrations of the samples and pooled 3 μl from each sample per 96‐well plate were pooled into a 0.65 ml Eppendorf tube. We used NEBNext Ultra II Library Prep Kit (New England BioLabs) to adapt the pools of 384 PCR products into Illumina sequencing libraries each with a unique 6 bp library index following the manufacturer's instructions. Library pool purification, quantification, and sequencing were previously described (Roffler et al., 2021). We demultiplexed raw sequence reads and clustered sample 100% similarity and used BLAST to assign prey items taxonomically against 12S vertebrate sequences in GenBank and from a custom 12S database of vertebrate tissue by the Levi Lab to fill gaps in Genbank. Taxonomic assignment, filtering, and quality control measures were carried out as previously described (Roffler et al., 2021).

### 3.4 Habitat characteristics

We estimated annual (all locations included) and denning season (April 15–July 31) home ranges for wolf packs using GPS‐collar data from 13 wolves captured and monitored during 2012–2018 (described in Roffler & Gregovich, 2018; Roffler et al., 2018). We used an autocorrelated kernel density estimator (AKDE; Fleming & Calabrese, 2017) implemented with the ctmm 0.3.2 package (Calabrese et al., 2016; Fleming & Calabrese, 2017) with R software (version 4.1; R Core Team, 2021). All home ranges were clipped to the shoreline. Three active dens did not have GPS‐collared wolves associated with them during the denning season, thus we used the median width of the denning season home range buffer around the den site of other GPS‐collar represented wolf packs (7534 m) to infer home range extent around these dens.

We quantified the habitat within (1) wolf denning season home ranges surrounding active den sites and (2) annual home ranges, by calculating the proportion of each landcover type and road density. Land cover was classified by tree size and stand density (Albert & Schoen, 2007; Caouette & DeGayner, 2005) into low‐, medium‐, and high‐volume old‐growth forests (volume measured the potential quantity of timber in board feet available per acre), young‐growth forests regenerating from clearcuts ≤25 and >25 years old, non‐forest vegetation (meadows, grasslands, and muskegs), and non‐vegetated areas (freshwater, brush, urban areas). Previous work demonstrated wolves on POW select low‐volume old‐growth forests (forests ≥150 years old containing the lowest density of large diameter trees in relation to medium‐ and high‐volume old‐growth) and young successional clearcuts (Roffler et al., 2018) as these two landcover categories are valuable to deer by providing high forage biomass (Alaback, 1982; Farmer & Kirchhoff, 2007). We quantified road densities within denning season home ranges by measuring the total distance of roads and dividing by the home range area. We classified roads into three categories: (1) closed (assumed inaccessible by highway vehicles; e.g., overgrown and narrow), (2) high clearance (assumed only accessible by high‐clearance vehicles; e.g., gravel logging roads), and (3) passenger (accessible to all highway vehicles; e.g., paved roads). Road GIS data were obtained from the Tongass National Forest roads with core attributes layer (https://gis.data.alaska.gov/datasets/usfs::tongass‐national‐forest‐roads‐with‐core‐attributes), and the non‐routed other roads layer covering private and non‐U.S. Forest Service lands in the Tongass (https://catalog.epscor.alaska.edu/dataset/non‐routed‐other‐roads). We quantified the proportion of each land cover type and density of roads within denning season home ranges area using the Tabulate Area tool in ArcMap 10.8.1 (ERSI).

We calculated metrics of habitat configuration and connectivity within wolf denning season and annual home ranges using FRAGSTATS 4.2 (McGarigal et al., 2012) and the landcover classes described above. We quantified the amount of edge between landcover classes per unit area (edge density; ED). To assess the degree of habitat type aggregation and subdivision we measured the number of patches (NP), and density of landcover patches (patches/km^2^; PD). We measured the degree of intermixing of habitat types using the FRAGSTATS metrics CONTAG (contagion), IJI (interspersion and juxtaposition index), PLADJ (proportion of like adjacencies), and COHESION (patch cohesion index). We also assessed patch diversity with Shannon's diversity index (SHDI) which measures the number of different patch types and the proportional distribution of area among patch types. We performed these analyses for each wolf denning season and annual home range at the landscape level including all landcover classes, then repeated the analyses for each landcover class separately.

### 3.5 Statistical analysis

We quantified wolf diet composition by calculating indices of the relative frequency of occurrence as (1) the occurrence per feces (O/F) index (the number of occurrences of a diet item divided by the total number of scat samples) and (2) the occurrence per item (O/I) index (the number of occurrences of a diet item divided by the total number occurrences of all diet items). We included O/F for comparison to previous wolf diet studies, but restricted statistical tests to O/I indices to avoid overcounting prey items that co‐occur in scats containing multiple species. We calculated indices of the relative frequency of occurrence of prey groups in wolf diets by wolf pack, by year, by wolf age at active den sites, and by home range type for each wolf pack. Because of the positive correlation between relative read abundance (RRA) and the biomass of prey consumed estimated from the volume per scat of undigested prey remains (Massey et al., 2021), we also quantified RRA. RRA was calculated as the proportion of prey DNA sequence reads in a scat sample divided by the total number of prey DNA sequences in that sample (Massey et al., 2021).

We tested the effects of season, year, wolf pack, wolf age, and wolf sex on diet composition (using the O/I index) with permutation‐based multivariate analysis of variance (PERMANOVA; Anderson, 2001) implemented in the adonis2 function in the vegan R package (Oksanen et al., 2016). We grouped consumed species or taxonomic units into diet item categories (Table S1), generated Jaccard dissimilarity matrices of the prey group presence/absence data and ran analyses with 9999 permutations. To identify diet items that contributed most to observed differences in wolf diets between groups (season, year, wolf pack, age, or sex), we used similarity percentage analysis (SIMPER; Clarke, 1993) with the vegan package using 9999 permutations and Kruskal–Wallis rank sum tests to assess differences in the contribution of prey items between groups.

We first conducted analyses using PERMANOVA including all wolf scat sample data to test the effect of season and year on diet composition measured using the O/I index. We used the estimated age of the scat and the date of collection to group scat samples into seasons (summer: May–September, winter: October–April), and defined year as the biological year (e.g., 2015 = May 1, 2015–April 30, 2016). We then used PERMANOVA to examine how wolf diet composition varied by wolf pack and year, including season (summer vs. winter) and home range type (denning vs. non‐denning) with pack and year in separate models. We created separate models for season and home range type because both variables break the biological year into temporal categories in different ways. We assigned scats collected within annual home ranges (defined above) to wolf packs and excluded scats that were not collected within a known wolf pack home range.

Second, we conducted PERMANOVA analyses to explore differences in diet composition measured using the O/I index of wolves associated with a den site versus diets during the remainder of the biological year. We differentiated between wolf scats collected at active den sites during denning season, and scats collected within that wolf pack's home range outside of the denning season. For scats to be included in the active den category they had to meet two criteria: (1) spatial (collected within 100 m of an active den site) and (2) temporal (collected during the time frame when wolves are associated with a den site and before they move to rendezvous sites, April 15–July 31). Non‐denning season scats were collected throughout the wolf pack's annual home range outside of the denning season (August 1–April 14). Finally, we restricted our analyses to only use data from wolf scats collected at active den sites and constructed models to test the effects of wolf pack and year on diet composition. We subsequently included wolf age (pup vs. adult, as determined by scat size) and wolf sex (determined genetically with the canid SRY marker) with wolf pack and year in separate models due to sample size limitations (age, pack, and biological year were independent variables in one model and sex, pack, and biological year were independent variables in a separate model).

We estimated diet diversity and specialization for wolves within their annual home range and wolves at active den sites, further subdividing wolves at active den sites by age class (pups vs. adults). We used Shannon's Diversity Index (*H′*; Shannon, 1948) which measures the diversity of species within a group (higher values indicate a more diverse community), and Levins' (1968) measure of niche breadth (*B*) which measures the degree of diet specialization (lower values indicate a narrow dietary niche, whereas higher values indicate greater dietary generalization). We also measured species richness (*S*) as the total number of wolf diet items identified per study site. We calculated individual‐based rarefaction curves using EstimateS 9.1.0 (Colwell, 2013) to determine if samples reached a species diversity asymptote (*H′*) indicating completeness of samples for comparison of wolf diet diversity among packs despite differences in sample size.

We tested the effects of the average relative frequency of deer in wolf pack diets and denning season home range characteristics (habitat composition, road density, and habitat configuration and connectivity metrics) on litter size using generalized linear models (GLMs) with Poisson distribution errors and a natural log function implemented using the glm function in the R stats package. Statistics were weighted using the weights.glm function based on the number of samples included for each pack and biological year sampled, as some packs were sampled for >1 biological year. We then tested the effects of (1) denning season home range characteristics on the average relative frequency of deer in wolf pack diets during the denning season and (2) annual home range characteristics on the average relative frequency of deer in wolf pack diets throughout the year (seasons pooled) using generalized linear models (GLMs) with Gaussian distribution errors, and statistics weighted for sample size using the weights.glm function. We first screened individual covariates for collinearity using a Pearson's correlation matrix and a threshold cutoff of *r* = .7 (Hosmer & Lemeshow, 2000). We used univariate logistic regression to identify informative covariates to include in the multivariate model and then developed a suite of models including all significant single variable models and additive combinations of significant covariates. We included all possible combinations of covariates due to their presumed biological relevance. Model selection was conducted using Akaike's Information Criterion corrected for small sample sizes (AICc). We considered the model with the lowest AICc value to be best supported and models with ΔAICc < 2 to be plausible (Burnham & Anderson, 2002). Covariate *β* estimates with 95% confidence intervals excluding 0 were considered significant to litter size or the relative proportion of deer in wolf diets (Burnham & Anderson, 2002).

## 4 RESULTS

### 4.1 Den monitoring.

During 2012–2020 we detected 13 active den sites, 11 of which were located on POW and two in Snow Pass. Seven of the POW wolf dens had nine GPS‐collared wolves associated with them. Active dens were located within the home ranges of nine wolf packs (Figure 1).

We collected 322 hair samples (2015: *n* = 1; 2016: *n* = 73, 2017: *n* = 125, 2018: *n* = 123) and 227 scat samples (2015: *n* = 13; 2016: *n* = 64, 2017: *n* = 98, 2018: *n* = 52) at active den sites. Of the scat samples, 114 were classified as originating from adult wolves (2015: *n* = 13; 2016: *n* = 32, 2017: *n* = 54, 2018: *n* = 15) and 113 from pups (2016: *n* = 32, 2017: *n* = 44, 2018: *n* = 37).

Minimum counts of pups at active den sites using individuals identified from genotyping hair or scat (described below), camera images, or visual observations ranged from 1 to 7 (Table 1), and mean litter size was 4 (SD = 2.13). Minimum counts of adults at active den sites ranged from 1 to 6 (mean = 2.7, SD = 1.43; Table 1).

**TABLE 1:** Active wolf dens and the number of wolves detected using noninvasive samples (hair and scat), motion‐detection camera images, and observations on Prince of Wales Island, Alaska, 2012–2020

Year	Pack	Den ID	DNA hair	DNA pup scat	DNA adult scat	Cameras	Observations	Minimum adults	Minimum pups
2012	Ratz	1					2 AD	2	
2012	Honker	2					2 AD	2	
2013	Staney	3				2 AD, 6 P		2	6
2014	Honker	4				3 AD, 3 P	4 AD, 7 P	4	7
2015	Honker	2	1F			5 AD	1 AD, 1 P	5	1
2016	Sandy Beach	5	1M, 1F	1F, 2M	1F	4 P		2	4
2016	Ratz	6	1F	3F, 3M	1F, 1M	6 P		2	6
2016	Staney	7		1F	1F, 1M	2 P		1	2
2016	Hydaburg	8	1F	3M	1F, 1M	2 AD, 5 P		2	5
2016	Snow Pass	9	2F			2 AD, 4 P		2	4
2017	Sandy Beach	10	1F, 1M	3F, 2M	1F, 1M	1 AD, 6 P	2 AD	2	6
2017	Old Franks	11	2F		3F, 1M	3 AD, 2 P	3 AD, 2 P	4	2
2017	Hydaburg	8	2F, 3M	2M	2F, 3M	4 AD, 5 P	1 AD	5	5
2017	Trocadero	12		1F, 2M	1F, 1M	4 AD	2 AD, 5 P	4	5
2017	Snow Pass	9	2F		2F, 1M	6 AD, 2 P		6	2
2018	Staney	3	2M	7F	2M	7 P	2 AD	2	7
2018	Old Franks	11	2F, 5M	2F, 2M	1F, 1M	4 AD, 4 P	3 AD, 1 P	4	4
2018	Hydaburg	8	1F	1M	1M		1 AD	1	1
2018	Honker	2	1F, 2M		2F	1 AD, 5 P	2 AD	2	5
2019	Staney	3					1 AD, 1 P	1	1
2020	Trocadero	12				2 AD, 1 P		2	1
2020	Snow Pass	13				2 AD, 6 P		2	6

*Note*: Minimum counts were the highest number of wolves observed using all methods.

Abbreviations: AD, adult (≥1 year old); F, female; M, male; P, pup (<1 year old).

### 4.2 Genotyping

The genotyping success rate (the percentage of samples that successfully amplified and passed quality control steps) of identifying individual wolves for all hair samples was 39% and 53% for all scat samples. We identified 65 wolves from hair and scat samples at active den sites. Twenty‐three wolves were identified from hair samples, five of which were detected in 2 consecutive years at the same den site, and one of which was detected for 3 consecutive years at the same den site. Twenty‐nine adult wolves were identified from scat samples, one of which was detected in consecutive years at the same den site. Thirty‐three wolf pups were detected from the scat samples, and none of the pups were subsequently detected in hair samples collected at the den sites during the same year, affirming the utility of individual genotyping wolf pups from scat samples. Three of the wolf pups detected from pup scat were identified at the same den site the following year from hair (*n* = 1), scat (*n* = 1), or both hair and scat (*n* = 1) samples. Fourteen wolves were identified from both hair and scat samples.

### 4.3 Diet composition

We collected 713 scat samples on Prince of Wales and surrounding islands (Figure 1) between 2014 and 2021 including during den monitoring and sampling periods throughout the year. After removing scats that originated from black bears (*n* = 13), amplification success rate of the scat samples used for diet analysis was 77%, thus 538 of the scats collected were included in subsequent analyses. Overall, the scat samples contained 35 diet items grouped into 12 categories (Table S1), and each sample contained 1–8 diet items (mean = 1.18, SD = 0.881). Individual‐based rarefactions curves for dietary diversity (*H′*) reached an asymptote between 15 and 20 samples (Figure S1).

Using data from all scat samples (*n* = 538) deer were the most frequently occurring prey item (O/I = 60.5%), followed by beaver (O/I = 15.8%), and black bear (O/I = 7.2%). Wolves also consumed birds (O/I = 4.5%), mustelids (O/I = 4.1%), salmon (O/I = 3.0%), and marine mammals (O/I = 1.9%) to a lesser extent (Table S1). Overall wolf diet composition calculated using the O/I index and RRA data revealed similar patterns (Table S1). Wolf diet composition varied significantly by year (*F* = 14.478, *p* = .001), but not by season (*F* = 0.831, *p* = .480). Season (summer vs. winter) was not a significant factor and therefore not used in subsequent wolf diet composition models.

**FIGURE 2:**
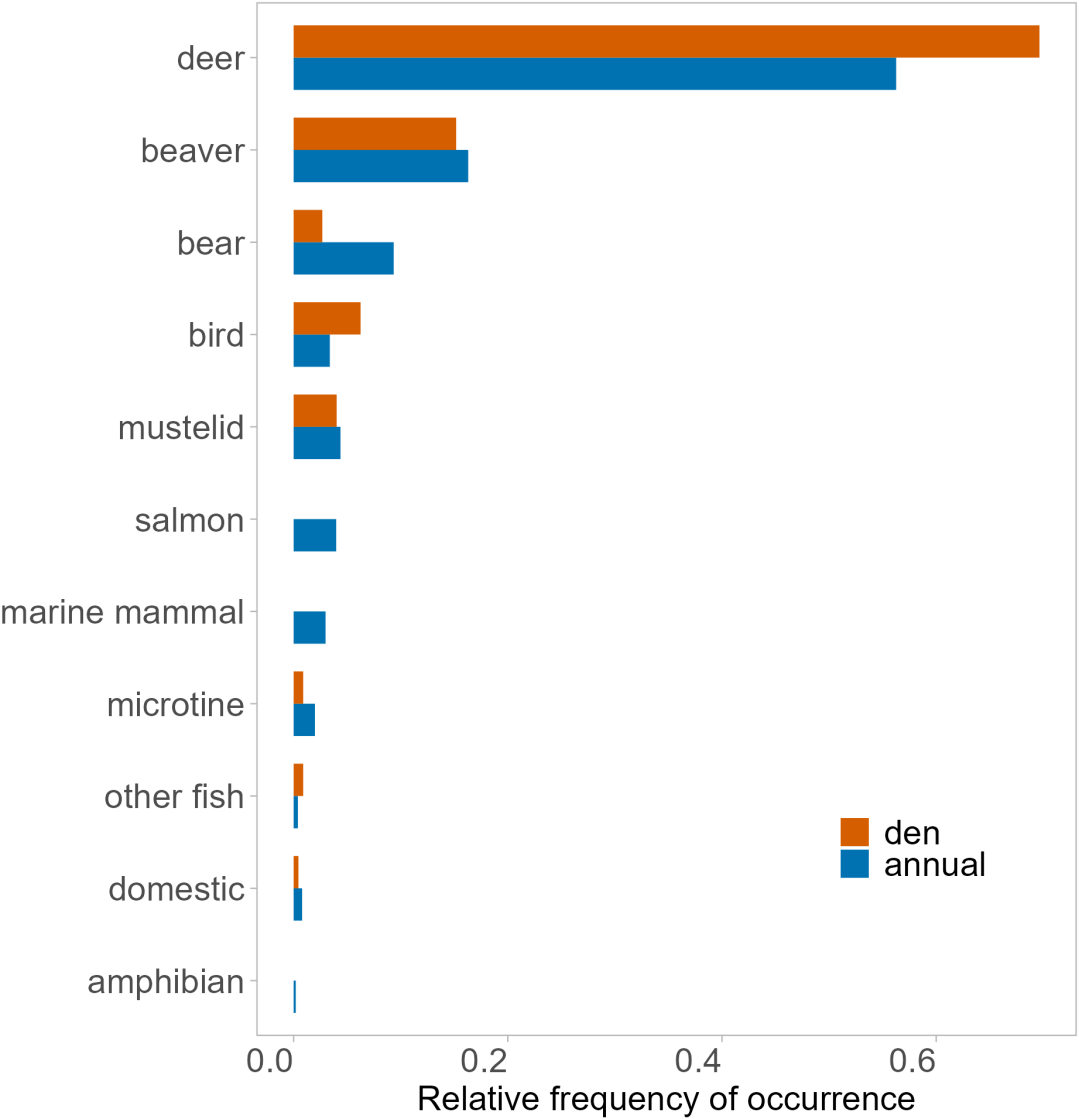
Diet composition of wolves based on the relative frequency (occurrence per item (O/I) index) of diet items identified in wolf scats (1) at active dens during the denning season (April 15–July 31; *n* = 178), and (2) throughout the wolf pack's annual home range outside of the denning season (August 1–April 14; *n* = 328), Prince of Wales Island, Alaska, 2015–2018.

**FIGURE 3:**
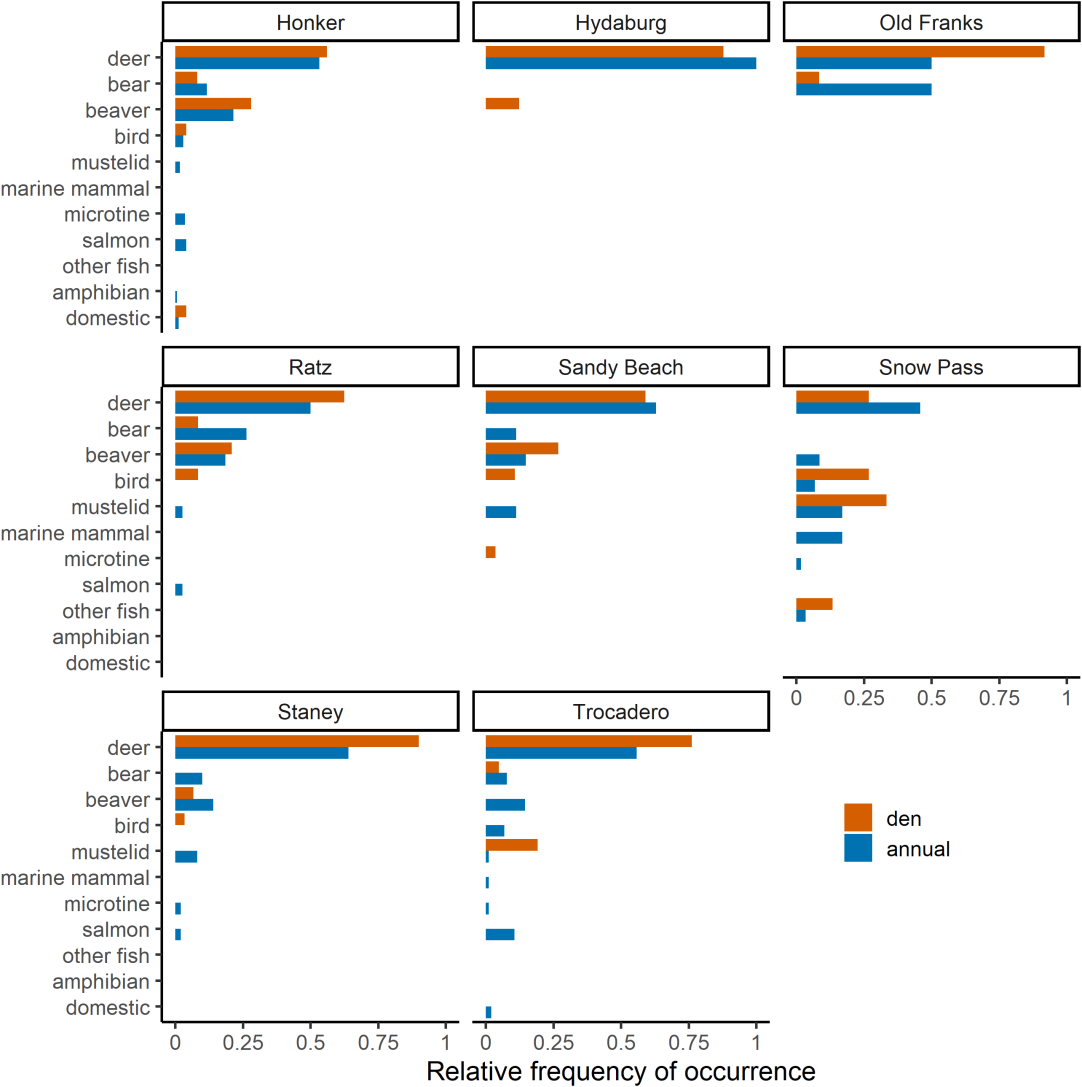
Diet composition by wolf pack based on the relative frequency (occurrence per item (O/I) index) of diet items identified in wolf scats (1) at active dens during the denning season (April 15–July 31), and (2) throughout the wolf pack's annual home range outside of the denning season (August 1–April 14), Prince of Wales Island, Alaska, 2015–2018.

We then considered the effects of wolf packs and home range type using samples collected within known wolf pack territories (*n* = 506). Diet composition varied significantly between wolves at den sites and within annual home ranges (*F* = 4.830, *p* = .007), wolf packs (*F* = 5.353, *p* = .001), and by year (*F* = 4.331, *p* = .004). Differences in diet composition by year were driven by contribution of important secondary prey species, specifically higher consumption of black bears during 2014 (O/I = 15.8%) and 2015 (O/I = 11.7%) than during 2016–2020 (O/I range = 2.8%–5.0%) and higher consumption of beaver during 2014–2016 (O/I range 18.8%–21.1%) than during 2017–2020 (O/I range = 0%–8.3%). SIMPER results showed dissimilarity in wolf diets at active den sites during denning season and wolf diets during non‐denning season was mostly influenced by variation in the contribution of beaver (11.7%), deer (9.8%), and black bear (4.6%), and black bear contribution to wolf diets was also identified as significantly different with Kruskal–Wallis tests (*X* ^2^ = 15.085, *df* = 1, *p* = .0001). Wolves at active den sites consumed more deer (O/I = 69.6%) than within their home range outside of denning season (O/I = 56.3%), and less black bear (denning season O/I = 2.7%; non‐denning season O/I = 9.3%; Figures 2 and 3). Wolf diet diversity was lowest (Shannon *H′* = 0.160) and niche breadth was narrowest (Levin's *B* = 1.961) during the denning season than during the non‐denning season (*H′* = 0.318; *B* = 2.817). Wolf diet species richness was also lower during the denning season (*S* = 15) than during the non‐denning season (*S* = 26).

**FIGURE 4:**
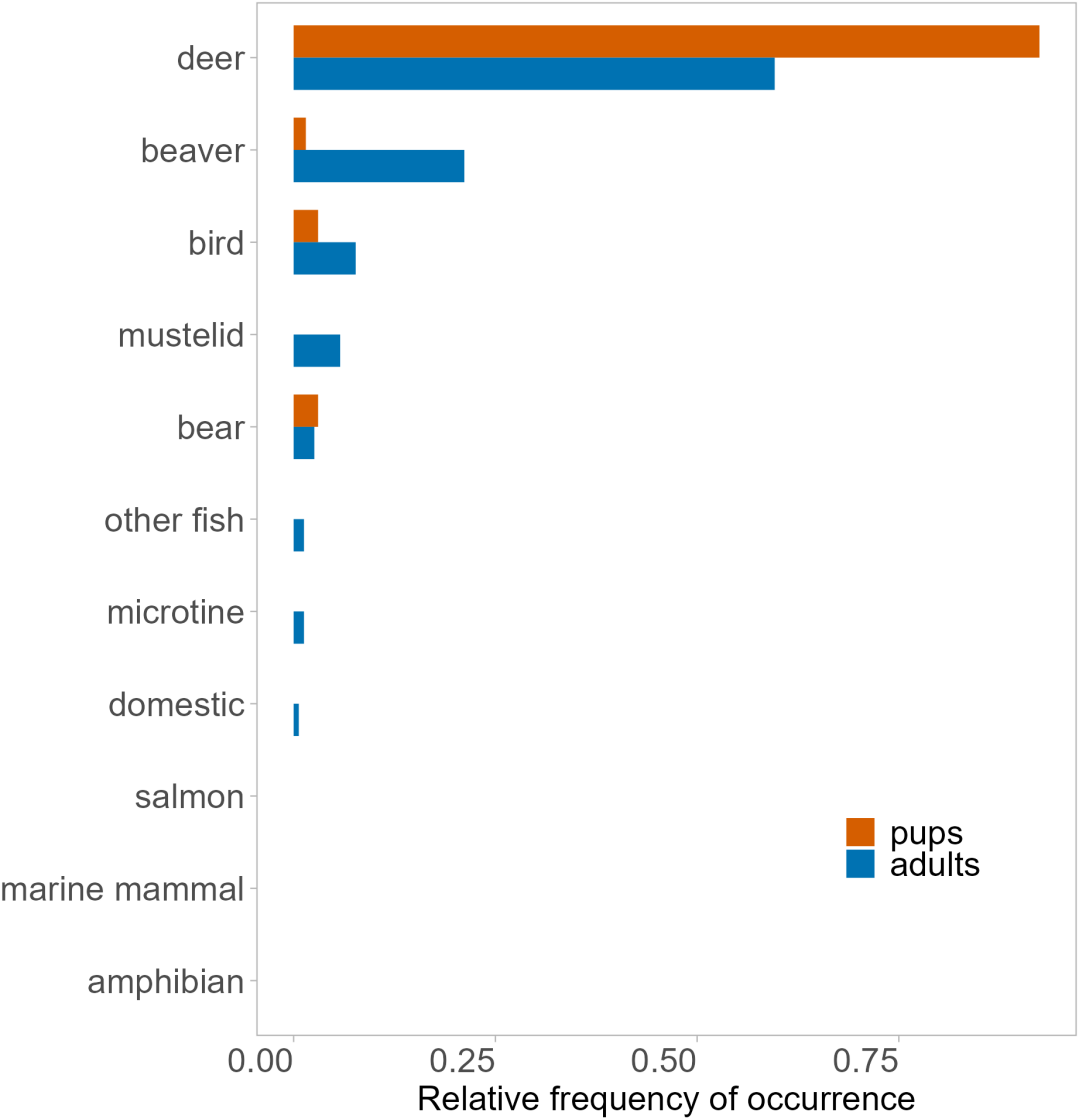
Diet composition of wolves based on the relative frequency (occurrence per item (O/I) index) of diet items identified in pup (*n* = 62) and adult (*n* = 114) wolf scats collected at active dens during the denning season (April 15–July 31), Prince of Wales Island, Alaska, 2015–2018.

Wolf diet composition determined from scats collected at active den sites (*n* = 176) varied by wolf pack (*F* = 6.804, *p* = .001) and by year (*F* = 3.4846, *p* = .025). When we included age‐specific diet information from pup (*n* = 62) and adult (*n* = 114) scats wolf diet composition differed by age class (*F* = 18.877, *p* = .001; Figure 4) and wolf pack (F = 5.352, *p* = .001; Figure S2). Overall dissimilarity between wolf pup and adult diets revealed by SIMPER was 31.8%, mainly driven by variation in the contribution of beaver (11.2%) and deer (9.8%). Beaver and deer consumption by wolf pups and adults was also significantly different quantified with Kruskal–Wallis tests (beaver: *X* ^2^ = 18.724, *df* = 1, *p* < .0001; deer: *X* ^2^ = 11.427, *df* = 1, *p* = .001). During denning season, pups consumed more deer (O/I = 92.4%) than adults (O/I = 59.1%), but less beaver (O/I = 1.5%) than adults (O/I = 21.4%). Wolf pup diet diversity was lower (*H′* = 0.044), and niche breadth was narrowest (Levin's *B* = 1.168) compared to adult diets (*H′* = 0.232; *B* = 2.505). Wolf pup diet species richness was also lower (*S* = 4) than adult diet species richness (*S* = 15).

We determined the sex of wolves from 63 scat samples (male = 26, female = 37); sex was not a significant factor in diet composition models.

### 4.4 Habitat characteristics and statistical analysis

Indices of dietary diversity (*B*, *H*′, *S*) were colinear and declined with increasing relative frequencies of deer in wolf diets (Roffler et al., 2021) and were thus excluded as independent variables in subsequent analyses. Non‐forest vegetation was colinear with young‐growth forest >25 years, passenger vehicle accessible roads (i.e., paved), and IJI, and was thus excluded. SHDI and CONTAG, PD and COHESION, and ED and PLADJ were colinear, so CONTAG, PD, and PLADJ were removed from subsequent analyses.

**FIGURE 5:**
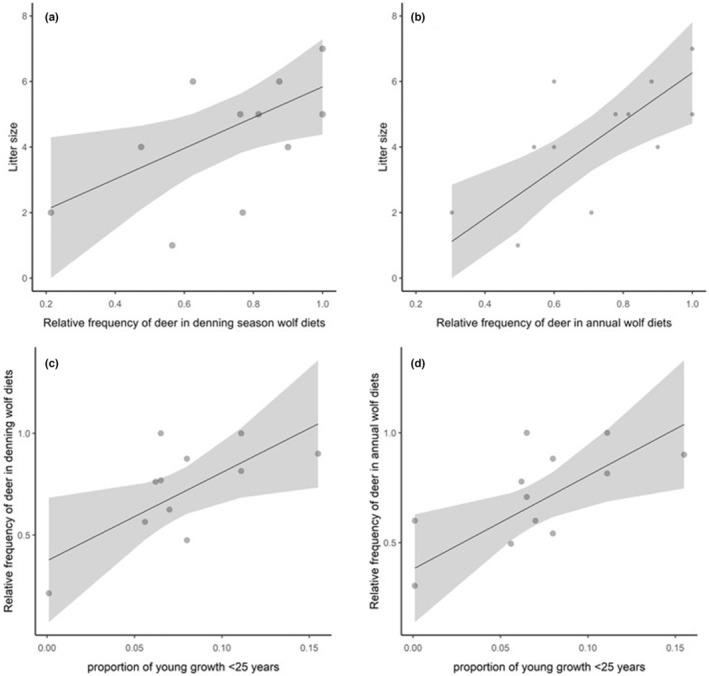
Litter size predicted by the relative frequency of deer in wolf diets (a) during denning season, and (b) annually, and the relative frequency of deer in wolf diets predicted by the proportion of young‐growth forest ≤25 years in (c) denning season home ranges and (d) annual home ranges, Prince of Wales Island, Alaska, 2015–2018. The lines indicate the fitted values of GLMs, with associated standard errors.

Litter size was only significantly influenced by the relative frequency of deer in wolf diets; no habitat covariates were significantly associated with litter size. Litter size was higher with increasing deer consumption during denning season (*β* = 1.126, SE = 0.173, *p* < .001; Figure 5a) and throughout the year (*β* = 1.914, SE = 0.143, *p* < .001; Figure 5b).

The relative frequency of deer in wolf diets during denning season was positively associated with the proportion of young‐growth forest ≤25 years old, IJI, SHDI, PR, and the density of closed roads within denning home ranges and negatively associated with the proportion of low‐volume old‐growth (Table 2). Five models had ΔAIC < 2; however, only the proportion of young‐growth forest ≤25 years old (*β* = 4.337, SE = 1.899, *p* = .048; Figure 5c) and IJI (*β* = 0.02, SE = 0.007, *p* = .021) coefficients did not have confidence intervals overlapping 0; therefore, the best supported model contained these two covariates (Table 2).

**TABLE 2:** Top‐ranked GLM models explaining variation in the relative frequency of deer in wolf diets during denning season and throughout the year influenced by home range habitat characteristics, Prince of Wales Island, Alaska, 2015–2020

	AICc	ΔAIC	*w*_*i*_
Denning season models			
YG ≤ 25 + LVPOG + IJI	−10.1	0	0.206
**YG ≤ 25 + IJI**	**−9.7**	**0.38**	**0.170**
YG ≤ 25 + LVPOG + closed roads + IJI	−9.4	0.71	0.144
YG ≤ 25 + LVPOG + IJI + SHDI	−8.3	1.85	0.082
YG ≤ 25 + IJI + SHDI	−8.1	1.96	0.077
Annual models			
YG YG ≤ 25 + closed roads	−7.5	0	0.337
**Closed roads**	**−7.2**	**0.33**	**0.287**
YG YG ≤ 25 + closed roads roads + ED	−5.8	1.72	0.143
**YG ≤ 25**	**−5.7**	**1.76**	**0.140**

*Note*: Akaike's information criterion (corrected for small sample size, “AICc”), ΔAICc, and AIC weight (“*w*_*i*_”) are shown. Models in bold were best supported. Model covariates are as follows: proportion of young‐growth less than 25 years old (YG ≤ 25), proportion of low volume old‐growth forest (LVPOG), interspersion and juxtaposition index (IJI), Shannon's diversity index (SHDI), edge density (ED), and density (km/km^2^) of closed roads (narrow, overgrown roads inaccessible by highway vehicles).

The relative frequency of deer in annual wolf diets was positively associated with the density of closed roads, the proportion of young‐growth forest ≤25 years old, and negatively associated with ED and within annual home ranges (Table 2). Although four models had ΔAIC < 2, only two univariate models containing density of closed roads, the proportion of young‐growth forest ≤25 years old were significant (*p* ≤ .05). Wolf consumption of deer throughout the biological year was positively influenced by the density of closed roads (*β* = 0.505, SE = 0.147, *p* = .006) and the proportion of young‐growth forest ≤25 years old (*β* = 4.249, SE = 1.656, *p* = .028; Figure 5d).

## 5 DISCUSSION

Wolf viability in our study system has been assumed to be linked to abundance of deer, the only ungulate occurring on POW, and this dependence of wolves on deer is a major basis for continued legal efforts to conserve old‐growth forests and manage young‐growth forests to maintain sustainable wolf‐deer predator–prey systems. In this study we found that the wolves sampled at the den site during mid‐May through mid‐July had a very narrow dietary niche breadth and the dominant prey species was deer, regardless of pack affiliation or year sampled. Deer consumption was also highest during the denning season relative to the rest of the year. High consumption of deer was also found in other studies that sampled at a similar scale (Bryan et al., 2006) or used comparable early pup‐rearing season data through mid‐July (Gable et al., 2018). Thus, despite the demonstrated consumption of a broad diversity of prey items on POW (Kohira & Rexstad, 1997, Massey et al., 2021; Roffler et al., 2021; Table S1) our results demonstrate a refinement of prey selection during the period when wolves are provisioning offspring and illustrate the reliance on deer during the early pup‐rearing phase.

The dominance of deer in wolf diets was even greater for pups than adults when separating age classes, and pups had a correspondingly narrow dietary niche in relation to adults. These results differ from Bryan et al. (2006) who also quantified adult and pup diets during den occupation and showed that deer consumption and dietary breadth were similar between age classes. Our results also diverge markedly from other work demonstrating the importance of smaller mammals to pups, especially beaver. In these studies, pups consumed beaver more frequently than adults (Gable et al., 2017; Mysłajek et al., 2019; Theberge & Cottrell, 1973), and in some cases beaver was the major diet item (O/F = 52%; Sidorovich et al., 2017). Beaver availability has been shown to be critical to pup survival by decreasing the risk of mortality from starvation (Benson et al., 2013), particularly when ungulates are scarce, or less vulnerable to predation as can be the case during summer (Forbes & Theberge, 1996; Fuller, 1989). In contrast, the contribution of beaver to POW wolf pups was minimal (O/I = 1.5%, O/F = 1.6%). Only four prey species were detected in wolf pups' diets throughout the course of the study (Figure 4), and at five of the nine active wolf dens, the only diet item detected in pup scats was deer (Figure S2). The low dietary diversity during denning season is particularly striking considering that DNA metabarcoding has been found to detect a greater variety of prey items and especially rare prey in comparison to mechanical sorting (Massey et al., 2021), which prior studies have used to characterize wolf diets. Because of this methodological advantage, we would expect to be able to detect rare prey items if they were being consumed by pups.

**FIGURE 6:**
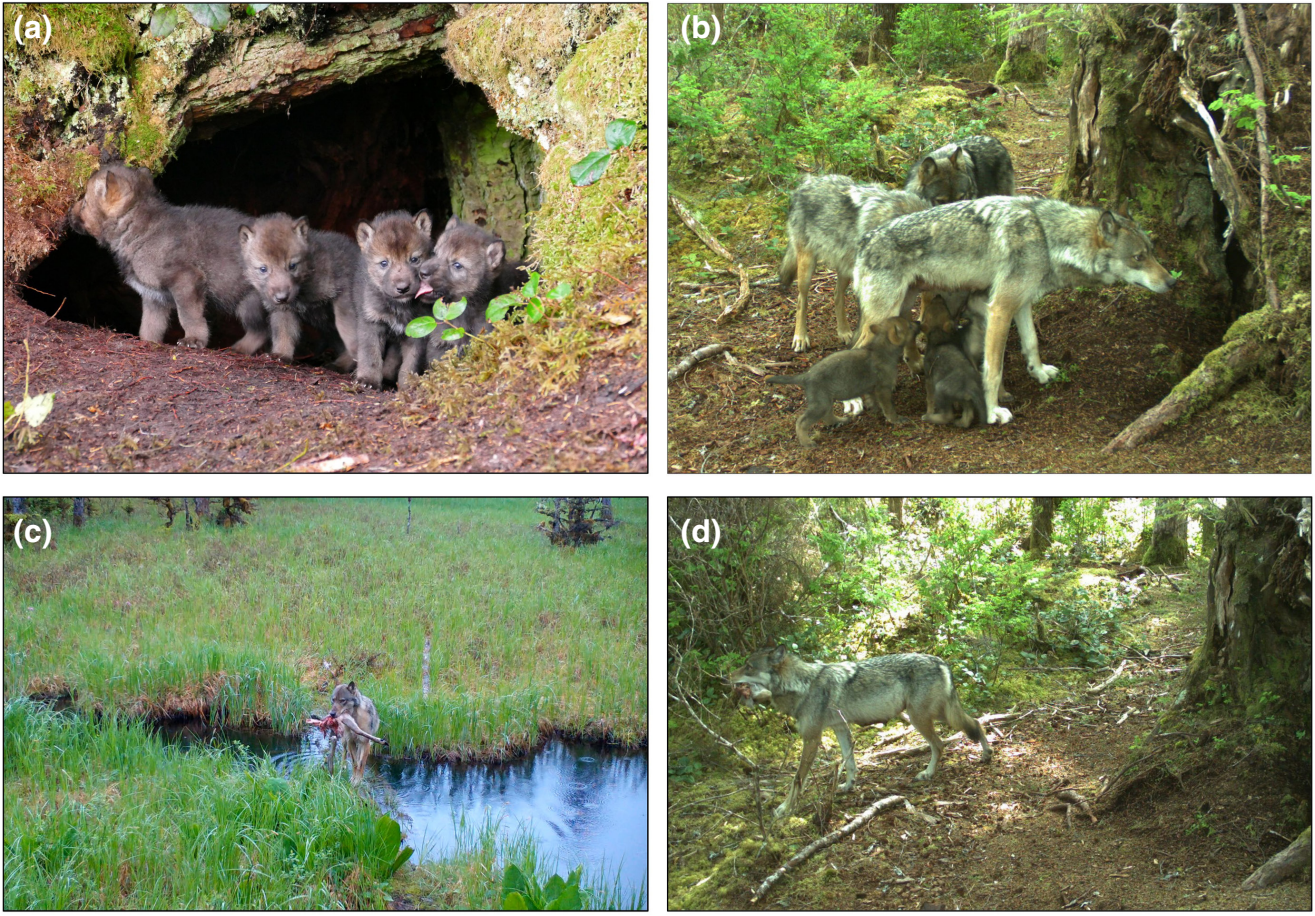
(a) and (b) Wolf pups and adults at active den sites, (c) wolf adult and (d) breeding female bringing portions of adult Sitka black‐tailed deer to active den sites, Prince of Wales Island, Alaska, 2015–2018. Photo credits (a), (b), and (d), ADF&G, (c) M. Kampnich.

That beaver played a relatively small role in the summer diets of wolf pups was inconsistent with it being the second most frequently consumed prey species by adults after deer (O/I = 21.4%). The dietary pattern of pups therefore cannot be explained by lack of beaver availability on POW, although annual consumption varied with lower contributions after 2016. Instead, differences in diet composition between pups and adults may be a result of selective provisioning as has been documented in other systems (Bryan et al., 2006). Providing pups with food of higher nutritional value or reduced parasitic burden is a strategy that may confer higher pup survival (Bryan et al., 2006). Alternatively, differences in adult and pup diets may be due to differences in the ease of transport and delivery of prey to den sites (Bryan et al., 2006). Sitka black‐tailed deer are relatively small (average weight adult male = 54 kg, average weight adult female = 36 kg; Schoen & Kirchhoff, 2016), and both observations and motion‐detecting cameras images have shown wolves carrying portions of adult deer to den sites (Figure 6), the remains of which we found during site investigations.

Measures of wolf dietary diversity on POW were lowest during the denning season relative to the rest of the year. These results contrast with other studies that showed more diverse wolf diets in the summer than in winter (Peterson & Ciucci, 2003; Spaulding et al., 1998) and which may also include a greater proportion of small‐bodied prey items (Mysłajek et al., 2019; Newsome et al., 2016; Stahler & Smith, 2006). Summer dietary diversity may increase as a result of wolves' restricted movements around den and rendezvous sites (Mech & Boitani, 2003; Newsome et al., 2016) in combination with lower availability or vulnerability of ungulates (Lodberg‐Holm et al., 2021; Paquet & Carbyn, 2003; Peterson & Ciucci, 2003; Spaulding et al., 1998).

The lower summer dietary diversity in our study system in comparison to previous research could potentially be explained by our focus spatially on den sites and temporally on the early pup‐rearing period when the wolf pack occupied these sites. In contrast, some other studies have characterized seasonal diets more broadly using data collected throughout the summer and included rendezvous sites and other sampling locations throughout the home range (Gable et al., 2018; Mysłajek et al., 2019; Sidorovich et al., 2017; Stahler & Smith, 2006; Steenweg et al., 2015). As wolves move from den sites to rendezvous sites during the pup‐rearing season, wolf diets can also shift due to changes in prey vulnerability. Neonate ungulates are often a key prey item during the pulse of births in late May and early June (Kunkel & Mech, 1994), but as fawns become less susceptible to predation in mid‐summer wolves may rely more on small prey items or even plants such as berries (Gable et al., 2018; Stahler & Smith, 2006). For example, although Gable et al. (2018) did not measure diets of wolves linked to den sites, they found dietary diversity sampled at the weekly scale was lowest at the beginning of their sampling period in late June and began to increase in late July.

We found that on POW increasing proportions of deer consumed by wolves had a positive association with litter size. The average litter size on POW during our study period (mean = 4, SD = 2.3) is comparable to values reported during 1993–2003 (mean = 4.1, SD = 1.7, Person & Russell, 2009) but smaller than the average across North American populations (5–6, Fuller et al., 2003). Variation in litter size is a key component of wolf reproduction and a function of the number of pups born and early pup survival. Because pups have limited mobility during the first few months of life and rely on adult members of the pack to deliver food (Packard, 2003), their survival hinges upon the success and efficiency of adults to acquire prey, which is influenced by prey availability within the pack's home range (Harrington et al., 1983; Van Ballenberghe & Mech, 1975). The amount and quality of food delivered to pups and subsequently consumed are fundamental to their growth and development (Fuller et al., 2003; Van Ballenberghe & Mech, 1975), and as pup survival is positively influenced by larger body size, steady sources of food to enhance body condition is vital (Van Ballenberghe & Mech, 1975). Prey abundance, and especially the abundance of ungulates that are available to adult wolves in the pack, is therefore important to their persistence because pup survival is directly related to ungulate biomass (Fuller et al., 2003). Indeed, previous research demonstrated both underweight pups (Van Ballenberghe & Mech, 1975) and decreased pup survival in areas of low ungulate abundance (Mech, 1977; Seal et al., 1975; Van Ballenberghe & Mech, 1975). Further, a positive correlation between litter size and available ungulate biomass has been found in study systems throughout North America (Boertje & Stephenson, 1992; Fuller, 1989; Fuller et al., 2003; Keith, 1983; Van Ballenberghe & Mech, 1975).

Although deer densities on POW have not been directly quantified at the landscape scale, abundance has been estimated in some watersheds with deer pellet transect surveys (McCoy, 2017) and by DNA mark‐recapture (Brinkman et al., 2011). Deer abundance with pellet transects have been estimated on POW since 1985 and indicated relatively high densities in comparison to other Southeast Alaskan islands and generally stable or increasing trends across most watersheds surveyed (mean number of pellet groups per plot range in 2015 = 1.05–2.27; McCoy, 2017). Deer abundance from DNA mark‐recapture demonstrated higher deer densities in old‐growth forests (12 deer/km^2^) and young‐growth forests that had been logged ≤30 years prior (10 deer/km^2^) than young‐growth >30 years old (7 deer/km^2^), indicating an important association with patterns of deer abundance and forest management (Brinkman et al., 2011). Early successional and open low‐volume old‐growth forests allow light penetration to the forest floor and promote growth of the shrubs and forbs preferred by deer, but during winter the lack of forest canopy allows snow to accumulate and not only potentially bury deer forage but also inhibit deer movement (Alaback, 1982; Kirchhoff & Schoen, 1987). Therefore, deer habitat quality assessments have focused on winter habitat as critical and the highest ranking habitats include high‐volume old‐growth forests, low elevation terrain, south facing aspects, and low snow levels (Gilbert et al., 2017; Shanley et al., 2021; Suring et al., 1992). Gilbert et al. (2017) found that during mild winters with low snow load deer selected young‐growth forests ≤30 years and avoided older young‐growth (>30 years old) and high volume old‐growth. However, the relationship was reversed during years with severe winters, indicating the habitat selection pattern for forests under different management and successional stages interacts with environmental factors (Gilbert et al., 2017). Young clearcuts provide ample regenerating understory shrubs to deer for forage, but the value of young‐growth forest to deer has been shown to decrease dramatically once passing the 25–30‐year threshold as stem‐exclusion reduces the amount of understory biomass (Alaback, 1982; Farmer & Kirchhoff, 2007), underscoring the importance of patterns of forest succession across the POW landscape.

In this study, we found an association with the amount of early successional young‐growth forest within both denning and annual wolf home ranges and the contribution of deer to wolf diets. The proportion of young‐growth ≤30 years varied among wolf home ranges from 0% to 16% of landcover (Tables S2 and S3). Although we expected that deer consumption would be driven partially by the availability of old‐growth forest due to its importance for deer, especially during winter (Kirchhoff & Schoen, 1987), we instead found no relationship with this habitat type and the contribution of deer to wolf diets. The lack of a clear relationship was likely exacerbated by the relatively mild winters on POW during our study period (National Oceanic and Atmospheric Administration, National Weather Service, 2022). Previous research on POW found no statistically significant difference in wolf consumption of deer in wolf home ranges that were unlogged compared to those that contained up to 26% of logged forest, although the age of the young‐growth forest resulting from logging was not specified (Kohira & Rexstad, 1997). In this study, nearly 30 years later, we did find a difference but only specific to young clearcuts that are expected to be selected by deer before stem‐exclusion.

Notably, young‐growth forests of all age classes were avoided by wolves within their denning season home ranges during 1995–2004 (Person & Russell, 2009) and during 2012–2016 (Roffler et al., 2018) in habitat selection models, even though this habitat type was ubiquitous. Outside of the pup‐rearing season wolves selected young‐growth ≤30 years old (Roffler et al., 2018), reflecting shifting preferences for habitat across seasons. It is possible that wolves chose to travel and hunt in areas in proximity to young clearcuts to increase their chances of encountering deer. Indeed, Farmer et al. (2006) found that on nearby Heceta Island (a 180 km^2^ island within the POW Island complex), the risk of wolf predation of adult and yearling female deer increased in young clearcuts, potentially due to the open landscape providing higher detection opportunities. Further, deer were more vulnerable to predation in fragmented habitats (Farmer et al., 2006), a result echoed by our current results indicating that deer consumption by wolves during denning season increased with higher levels of IJI (interspersion and juxtaposition index). This metric of habitat configuration describes the intermixing and adjacency of a habitat patch type to other patch types within landscape; well mixed patches result in high IJI values, and low patch type mixing results in low IJI values (McGarigal et al., 2012). Therefore, because deer occur at higher densities in old‐growth and young‐growth ≤30 years old (Brinkman et al., 2011), but are more vulnerable in young and open clearcuts (Farmer et al., 2006), the fragmented nature of POW forests may promote detection and acquisition of deer by wolves.

The density of closed roads within the annual home range was positively associated with the relative proportion of deer in annual wolf diets, but not a significant factor during the denning season. These results mirror our previous seasonal habitat selection analyses which indicated wolves on POW strongly selected areas of high road densities during winter but avoided roads during the denning season (Roffler et al., 2018). Roads may be a more important feature to increase efficiency of wolf movement and prey encounters during fall and winter when space use across the pack home range is more widespread (Dickie et al., 2022; Houle et al., 2010; Lesmerises et al., 2013), and territorial behavior requires greater movement (Jedrzejewski et al., 2001) in comparison to the pup‐rearing period when wolves have more restricted activity centers (Benson et al., 2015; Houle et al., 2010; Person & Russell, 2009; Theuerkauf, 2009). Importantly, roads may promote faster movement rates and travel efficiency for wolves (Dickie et al., 2022; Finnegan et al., 2018; Pigeon et al., 2020) which may result in increased prey encounter and kill rates (Dickie et al., 2017; Zimmermann et al., 2014). The availability of closed roads (narrow, overgrown roads inaccessible by highway vehicles) was an important factor for increasing deer consumption, whereas roads that would be accessible to vehicles (high‐clearance and passenger) were not. Wolves may be focusing on using closed rather than open, high traffic roads for travel and access to high quality deer habitat resulting in an increase in the proportion of deer in wolf diets.

The benefits of early successional vegetation in young‐growth forest to deer has a limited time frame post‐logging, and deer abundance is predicted to decline as a greater proportion of the young‐growth forests on POW move into the stem‐exclusion phase (Alaback, 1982; Farmer & Kirchhoff, 2007; Person, 2001). As deer are the primary prey of wolves on POW and in many areas of Southeast Alaska, this presents the question of whether wolves may be able to switch to other prey if deer were to become less available. Our recent work indicates that wolves in this region responded to lower dietary contributions of their primary ungulate prey by increasing the diversity of prey consumed (Roffler et al., 2021), suggesting wolves could tolerate large‐scale ecological changes resulting in decreased abundance of deer. Although wolves are highly adaptable and display dietary plasticity (Peterson & Ciucci, 2003), which is favorable to ensuring their persistence to environmental change and shifts in prey abundance and composition, other modeling efforts have pointed to how decreased deer habitat and abundance may be detrimental to wolf population growth rates and may trigger population declines (Gilbert et al., 2022; Person, 2001). Here we provide evidence of a possible adverse effect of deer declines to wolf fitness by linking the contribution of deer in wolf diets to litter size. Although our sample size is limited, and further work would be valuable to gain a deeper understanding of the influence of habitat and prey availability on wolf population viability, we documented the prime importance of deer to components of wolf reproduction and fitness. Our results suggest that one possible outcome of landscape‐level reductions in deer habitat capability and abundance could be reduced wolf litter sizes and a corresponding decrease in the wolf population. However, considering the ample availability of alternate prey on POW, it is likely wolves would persist albeit at lower densities.

## AUTHOR CONTRIBUTIONS

**Gretchen H. Roffler:** Conceptualization (lead); data curation (equal); formal analysis (lead); funding acquisition (lead); investigation (lead); methodology (supporting); project administration (lead); writing – original draft (lead); writing – review and editing (lead). **Kristine L. Pilgrim:** Data curation (equal); formal analysis (supporting); methodology (supporting); writing – review and editing (supporting). **Katherine E. Zarn:** Formal analysis (supporting); investigation (supporting); methodology (supporting); writing – review and editing (supporting). **Michael K. Schwartz:** Resources (supporting); writing – review and editing (supporting). **Taal Levi:** Data curation (lead); formal analysis (supporting); methodology (lead); resources (lead); validation (lead); writing – review and editing (supporting).

## CONFLICT OF INTEREST

The authors declare that they have no known competing financial interests or personal relationships that could have appeared to influence the work reported in this paper.

## Supporting information


AppendixS1

## Data Availability

The data supporting the study are available at the Dryad Digital Repository https://doi.org/10.5061/dryad.cjsxksn9g.

## References

[ece39648-bib-0001] Alaback, P. B. (1982). Dynamics of understudy biomass in Sitka spruce‐western hemlock forests of Southeast Alaska. Ecology, 63(6), 1932–1948.

[ece39648-bib-0002] Albert, D. M. , & Schoen, J. W. (2007). A conservation assessment for the coastal forests and mountains ecoregion of southeastern Alaska and the Tongass National Forest. In J. W. Schoen & E. Dovichin (Eds.), A conservation assessment and resource synthesis for the coastal forests and mountains ecoregion in Southeastern Alaska and the Tongass National Forest. Audubon Alaska and The Nature Conservancy.

[ece39648-bib-0003] Albert, D. M. , & Schoen, J. W. (2013). Use of historical logging patterns to identify disproportionately logged ecosystems within temperate rainforests of southeastern Alaska. Conservation Biology, 27(4), 774–784. 10.1111/cobi.12109 23866037

[ece39648-bib-0004] Anderson, M. J. (2001). A new method for non‐parametric multivariate analysis of variance. Austral Ecology, 26(1), 32–46. 10.1111/j.1442-9993.2001.tb00081.x

[ece39648-bib-0005] Benson, J. F. , Mills, K. J. , Loveless, K. M. , & Patterson, B. R. (2013). Genetic and environmental influences on pup mortality risk for wolves and coyotes within a Canis hybrid zone. Biological Conservation, 166, 133–141. 10.1016/j.biocon.2013.06.018

[ece39648-bib-0006] Benson, J. F. , Mills, K. J. , & Patterson, B. R. (2015). Resource selection by wolves at dens and rendezvous sites in Algonquin park, Canada. Biological Conservation, 182, 223–232. 10.1016/j.biocon.2014.12.010

[ece39648-bib-0007] Boertje, R. D. , & Stephenson, R. O. (1992). Effects of ungulate availability on wolf reproductive potential in Alaska. Canadian Journal of Zoology, 70(1967), 2441–2443. 10.1139/z92-328

[ece39648-bib-0008] Brinkman, T. J. , Person, D. K. , Chapin, F. S. , Smith, W. , & Hundertmark, K. J. (2011). Estimating abundance of Sitka black‐tailed deer using DNA from fecal pellets. The Journal of Wildlife Management, 75(1), 232–242. 10.1002/jwmg.22

[ece39648-bib-0009] Bryan, H. M. , Darimont, C. T. , Reimchen, T. E. , & Paquet, P. C. (2006). Early ontogenetic diet in Gray Wolves, *Canis lupus*, of coastal British Columbia. The Canadian Field‐Naturalist, 120(1), 61–66. 10.22621/cfn.v120i1.247

[ece39648-bib-0010] Burnham, K. P. , & Anderson, D. R. (2002). Model selection and multi‐ model inference: A practical information‐theoretic approach (2nd ed.). Springer‐Verlag.

[ece39648-bib-0011] Calabrese, J. M. , Fleming, C. H. , & Gurarie, E. (2016). Ctmm: An R package for analyzing animal relocation data as a continuous‐time stochastic process. Methods in Ecology and Evolution, 7(9), 1124–1132. 10.1111/2041-210X.12559

[ece39648-bib-0012] Caouette, J. P. , & DeGayner, E. J. (2005). Predictive mapping for tree sizes and densities in southeast Alaska. Landscape and Urban Planning, 72(1–3), 49–63. 10.1016/j.landurbplan.2004.09.012

[ece39648-bib-0013] Ciucci, P. , Boitani, L. , Pelliccioni, E. R. , Rocco, M. , & Guy, I. (1996). A comparison of scat‐analysis methods to assess the diet of the wolf. Wildlife Biology, 2(1), 37–48. 10.1002/etc.142

[ece39648-bib-0014] Clarke, K. R. (1993). Non‐parametric multivariate analyses of changes in community structure. Australian Journal of Ecology, 18(1), 117–143. 10.1111/j.1442-9993.1993.tb00438.x

[ece39648-bib-0015] Colwell, R. K. (2013). EstimateS, Version 9.1: Statistical estimation of species richness and shared species from samples (Software and User's Guide) . Available at: http://purl.oclc.org/estimates

[ece39648-bib-0016] Darimont, C. T. , Price, M. H. H. , Winchester, N. N. , Gordon‐Walker, J. , & Paquet, P. C. (2004). Predators in natural fragments: Foraging ecology of wolves in British Columbia's central and north coast archipelago. Journal of Biogeography, 31, 1867–1877.

[ece39648-bib-0017] Dickie, M. , Serrouya, R. , Avgar, T. , McLoughlin, P. , McNay, R. S. , DeMars, C. , Boutin, S. , & Ford, A. T. (2022). Resource exploitation efficiency collapses the home range of an apex predator. Ecology, 103(5), e3642. 10.1002/ecy.3642 35066867

[ece39648-bib-0018] Dickie, M. , Serrouya, R. , McNay, R. S. , & Boutin, S. (2017). Faster and farther: Wolf movement on linear features. Journal of Applied Ecology, 54(1), 253–263. 10.1111/1365-2664.12732

[ece39648-bib-0019] Dorendorf, R. (2021). Survey Memo: GMU 2 Wolf Population Estimate, Fall 2020. Alaska Department of Fish and Game.

[ece39648-bib-0020] Duchamp, C. , Boyer, J. , Briaudet, P. E. , Leonard, Y. , Moris, P. , Bataille, A. , Dahier, T. , Delacour, G. , Millischer, G. , Miquel, C. , Poillot, C. , & Marboutin, E. (2012). A dual frame survey to assess time‐ and space‐related changes of the colonizing wolf population in France. Hystrix, 23(1), 1–12. 10.4404/hystrix-23.1-4559

[ece39648-bib-0021] Farmer, C. J. , & Kirchhoff, M. D. (2007). Ecological classifications of deer habitat in the Tongass National Forest, Alaska. Northwestern Naturalist, 88(2), 73–84.

[ece39648-bib-0022] Farmer, C. J. , Person, D. K. , & Bowyer, R. T. (2006). Risk factors and mortality of black‐tailed deer in a managed forest landscape. Journal of Wildlife Management, 70(5), 1403–1415. 10.2193/0022-541X(2006)70

[ece39648-bib-0023] Finnegan, L. , Pigeon, K. E. , Cranston, J. , Hebblewhite, M. , Musiani, M. , Neufeld, L. , Schmiegelow, F. , Duval, J. , & Stenhouse, G. B. (2018). Natural regeneration on seismic lines influences movement behaviour of wolves and grizzly bears. PLoS ONE, 13(4), e0195480. 10.1371/journal.pone.0195480 29659615 PMC5901995

[ece39648-bib-0024] Fleming, C. H. , & Calabrese, J. M. (2017). A new kernel density estimator for accurate home‐range and species‐range area estimation. Methods in Ecology and Evolution, 8(5), 571–579. 10.1111/2041-210X.12673

[ece39648-bib-0025] Forbes, G. J. , & Theberge, J. B. (1996). Response by wolves to prey variation in central Ontario. Canadian Journal of Zoology, 74(8), 1511–1520. 10.1139/z96-165

[ece39648-bib-0026] Francisco, L. V. , Langston, A. A. , Mellersh, C. S. , Neal, C. L. , & Ostrander, E. A. (1996). A class of highly polymorphic tetranucleotide repeats for canine genetic mapping. Mammalian Genome, 7, 359–362. 8661717 10.1007/s003359900104

[ece39648-bib-0027] Fredholm, M. , & Wintero, A. K. (1995). Variation of short tandem repeats within and between species belonging to the Canidae family. Mammalian Genome, 6, 11–18. 7719020 10.1007/BF00350887

[ece39648-bib-0028] Fuller, T. K. (1989). Population dynamics of wolves in North‐Central Minnesota. Wildlife Monographs, 105, 3–41.

[ece39648-bib-0029] Fuller, T. K. , Mech, L. D. , & Cochrane, J. F. (2003). Wolf population dynamics. In L. D. Mech & L. Boitani (Eds.), Wolves: Behavior, ecology, and conservation (pp. 161–191). University of Chicago Press. 10.1016/j.jmr.2013.02.009

[ece39648-bib-0030] Gable, T. D. , Windels, S. K. , & Bruggink, J. G. (2017). The problems with pooling poop: Confronting sampling method biases in wolf (*Canis lupus*) diet studies. Canadian Journal of Zoology, 95(11), 843–851. 10.1139/cjz-2016-0308

[ece39648-bib-0031] Gable, T. D. , Windels, S. K. , Bruggink, J. G. , & Barber‐Meyer, S. (2018). Weekly summer diet of Gray Wolves (*Canis lupus*) in Northeastern Minnesota. The American Midland Naturalist, 179(1), 15–27. 10.1016/j.tig.2014.09.010

[ece39648-bib-0032] Galpern, P. , Manseau, M. , Hettinga, P. , Smith, K. , & Wilson, P. (2012). Allelematch: An R package for identifying unique multilocus genotypes where genotyping error and missing data may be present. Molecular Ecology Resources, 12, 771–778. 10.1111/j.1755-0998.2012.03137.x 22463778

[ece39648-bib-0033] Gilbert, S. L. , Haynes, T. , Lindberg, M. S. , Albert, D. M. , Kissling, M. , Lynch, L. , & Person, D. (2022). Potential futures for coastal wolves and their ecosystem services in Alaska, with implications for management of a social‐ecological system. Frontiers in Ecology and Evolution, 10, 809371. 10.3389/fevo.2022.809371

[ece39648-bib-0034] Gilbert, S. L. , Hundertmark, K. J. , Person, D. K. , Lindberg, M. S. , & Boyce, M. S. (2017). Behavioral plasticity in a variable environment: Snow depth and habitat interactions drive deer movement in winter. Journal of Mammalogy, 98(1), 246–259. 10.1093/jmammal/gyw167

[ece39648-bib-0035] Harrington, F. H. , Mech, L. D. , & Fritts, S. H. (1983). Pack size and wolf pup survival: Their relationship under varying ecological conditions. Behavioral Ecology and Sociobiology, 13(1), 19–26.

[ece39648-bib-0036] Holmes, N. G. , Dickens, H. F. , Parker, H. L. , Binns, M. M. , Mellersh, C. S. , & Sampson, J. (1995). Eighteen canine microsatellites. Animal Genetics, 25, 132–133. 10.1111/j.1365-2052.1995.tb02659.x 7733507

[ece39648-bib-0037] Hosmer, D. W. , & Lemeshow, S. (2000). Applied logistic regression (2nd ed.). John Wiley and Sons.

[ece39648-bib-0038] Houle, M. , Fortin, D. , Dussault, C. , Courtois, R. , & Ouellet, J. P. (2010). Cumulative effects of forestry on habitat use by gray wolf (*Canis lupus*) in the boreal forest. Landscape Ecology, 25(3), 419–433. 10.1007/s10980-009-9420-2

[ece39648-bib-0039] Jedrzejewski, W. , Schmidt, K. , Theuerkauf, J. , Jedrzejewska, B. , & Okarma, H. (2001). Daily movements and territory use by radio‐collared wolves (*Canis lupus*) in Bialowieza Primeval Forest in Poland. Canadian Journal of Zoology, 79, 1993–2004. 10.1139/cjz-79-11-1993

[ece39648-bib-0040] Keith, L. B. (1983). Population dynamics of wolves. In L. N. Carbyn (Ed.), Wolves in Canada and Alaska: Their status, biology, and management. Report Series number 45 (pp. 66–77). Canadian Wildlife Service.

[ece39648-bib-0041] Kirchhoff, M. D. , & Schoen, J. W. (1987). Forest cover and snow: Implications for deer habitat in southeast Alaska. Journal of Wildlife Management, 51(1), 28–33. 10.2307/3801623

[ece39648-bib-0042] Kohira, M. , & Rexstad, E. A. (1997). Diets of wolves, *Canis lupus*, in logged and unlogged forests of southeastern Alaska. Canadian Field Naturalist, 111(3), 429–435.

[ece39648-bib-0043] Kunkel, K. E. , & Mech, L. D. (1994). Wolf and bear predation on white‐tailed deer fawns in northeastern Minnesota. Canadian Journal of Zoology, 72(9), 1557–1565.

[ece39648-bib-0044] Lesmerises, F. , Dussault, C. , & St‐Laurent, M. H. (2013). Major roadwork impacts the space use behaviour of gray wolf. Landscape and Urban Planning, 112(1), 18–25. 10.1016/j.landurbplan.2012.12.011

[ece39648-bib-0045] Levins, R. (1968). Toward an evolutionary theory of the niche. In E. T. Drake (Ed.), Evolution and environment (pp. 325–340). Yale University Press.

[ece39648-bib-0046] Lodberg‐Holm, H. K. , Teglas, B. S. , Tyers, D. B. , Jimenez, M. D. , & Smith, D. W. (2021). Spatial and temporal variability in summer diet of gray wolves (*Canis lupus*) in the Greater Yellowstone Ecosystem. Journal of Mammalogy, 102(4), 1030–1041. 10.1093/jmammal/gyab060 34393668 PMC8362331

[ece39648-bib-0047] Massey, A. , Roffler, G. H. , Vermeul, T. , Allen, J. M. , & Levi, T. (2021). Comparison of mechanical sorting and DNA metabarcoding for diet analysis with degraded wolf scats. Ecosphere, 12(6), e03557. 10.1101/2019.12.13.875898

[ece39648-bib-0048] McCoy, K. (2017). Sitka black‐tailed deer pellet‐group surveys in Southeast Alaska, 2016 report . Alaska Department of Fish and Game, Wildlife Management Report ADF&G/DWC/WMR2017‐2 (Juneau, Alaska).

[ece39648-bib-0049] McGarigal, K. , Cushman, S. A. , & Ene, E. (2012). FRAGSTATS v4: Spatial pattern analysis program for categorical and continuous maps . Computer software program produced by the authors at the University of Massachusetts, Amherst. Available at: http://www.umass.edu/landeco/research/fragstats/fragstats.html

[ece39648-bib-0050] McKelvey, K. S. , & Schwartz, M. K. (2005). Dropout: A program to identify problem loci and samples for noninvasive genetic samples in a capture‐mark‐recapture framework. Molecular Ecology Notes, 5(3), 716–718. 10.1111/j.1471-8286.2005.01038.x

[ece39648-bib-0051] Mech, L. D. (1977). Productivity, mortality, and population trends of wolves in Northeastern Minnesota. Journal of Mammalogy, 58(4), 559–574. 10.2307/1380004

[ece39648-bib-0052] Mech, L. D. , & Boitani, L. (2003). Wolf social ecology. In L. D. Mech & L. Boitani (Eds.), Wolves: Behaviour, ecology, and conservation (pp. 1–34). University of Chicago Press.

[ece39648-bib-0053] Mech, L. D. , & Peterson, R. O. (2003). Wolf‐prey relations. In L. D. Mech & L. Boitani (Eds.), Wolves: Behaviour, ecology, and conservation (pp. 131–160). University of Chicago Press.

[ece39648-bib-0054] Mysłajek, R. W. , Tomczak, P. , Tołkacz, K. , Tracz, M. , Tracz, M. , & Nowak, S. (2019). The best snacks for kids: The importance of beavers Castor fiber in the diet of wolf *Canis lupus* pups in north‐western Poland. Ethology Ecology and Evolution, 31(6), 506–513. 10.1080/03949370.2019.1624278

[ece39648-bib-0055] National Oceanic and Atmospheric Administration, National Weather Service . (2022). Online weather data . Retrieved March 1, 2022, from https://w2.weather.gov/climate/xmacis.php?wfo=pajk

[ece39648-bib-0056] Newsome, T. M. , Boitani, L. , Chapron, G. , Ciucci, P. , Dickman, C. R. , Dellinger, J. A. , López‐Bao, J. V. , Peterson, R. O. , Shores, C. R. , Wirsing, A. J. , & Ripple, W. J. (2016). Food habits of the world's grey wolves. Mammal Review, 46(4), 255–269. 10.1111/mam.12067

[ece39648-bib-0057] Oksanen, J. , Blanchet, G. , Kindt, R. , Legendre, P. , Minchin, P. R. , O’Hara, R. B. , Simpson, G. L. , Solymos, P. , Stevens, M. H. H. , & Wagner, H. (2016). vegan: Community ecology package .

[ece39648-bib-0058] Ostrander, E. A. , Sprague, G. F. , & Rine, J. (1993). Identification and characterization of dinucleotide repeat (CA) in markers for genetic mapping in dog. Genomics, 16, 207–213. 8486359 10.1006/geno.1993.1160

[ece39648-bib-0059] Packard, J. M. (2003). Wolf behavior: Reproductive, social, and intelligent. In L. D. Mech & L. Boitani (Eds.), Wolves: Behavior, ecology and conservation (pp. 35–65). University of Chicago Press.

[ece39648-bib-0060] Paquet, P. C. , & Carbyn, L. N. (2003). Gray Wolf: *Canis lupus* and Allies. In G. A. Feldhammer , B. C. Thompson , & J. A. Chapman (Eds.), Wild mammals of North America: Biology, management, and conservation (2nd ed., pp. 482–510). John Hopkins University Press.

[ece39648-bib-0061] Peakall, R. , & Smouse, P. E. (2012). GenALEx 6.5: Genetic analysis in Excel. Population genetic software for teaching and research‐an update. Bioinformatics, 28(19), 2537–2539. 10.1093/bioinformatics/bts460 22820204 PMC3463245

[ece39648-bib-0062] Person, D. K. (2001). Alexander archipelago wolves: Ecology and population viability in a disturbed, insular landscape. University of Alaska Fairbanks.

[ece39648-bib-0063] Person, D. K. , & Russell, A. L. (2009). Reproduction and den site selection by wolves in a disturbed landscape. Northwest Science, 83(3), 211–224.

[ece39648-bib-0064] Peterson, R. O. , & Ciucci, P. (2003). The wolf as a Carnivore. In L. D. Mech & L. Boitani (Eds.), Wolves: Behavior, ecology, and conservation (pp. 104–130). University of Chicago Press.

[ece39648-bib-0065] Pigeon, K. E. , MacNearney, D. , Hebblewhite, M. , Musiani, M. , Neufeld, L. , Cranston, J. , Stenhouse, G. , Schmiegelow, F. , & Finnegan, L. (2020). The density of anthropogenic features explains seasonal and behaviour – Based functional responses in selection of linear features by a social predator. Scientific Reports, 10(1), 11437. 10.1038/s41598-020-68151-7 32651419 PMC7351780

[ece39648-bib-0066] Piry, S. , Alapetite, A. , Cornuet, J.‐M. , Paetkau, D. , Baudouin, L. , & Estoup, A. (2004). GENECLASS2: A software for genetic assignment and first‐generation migrant detection. The Journal of Heredity, 95(6), 536–539. 10.1093/jhered/esh074 15475402

[ece39648-bib-0067] R Core Team . (2021). R: A language and environment for statistical computing. R Foundation for Statistical Computing https://www.R‐project.org/

[ece39648-bib-0068] Riaz, T. , Shehzad, W. , Viari, A. , Pompanon, F. , Taberlet, P. , & Coissac, E. (2011). EcoPrimers: Inference of new DNA barcode markers from whole genome sequence analysis. Nucleic Acids Research, 39(21), 1–11. 10.1093/nar/gkr732 21930509 PMC3241669

[ece39648-bib-0069] Roffler, G. H. , Allen, J. M. , Massey, A. , & Levi, T. (2021). Metabarcoding of fecal DNA shows dietary diversification in wolves substitutes for ungulates in an island archipelago. Ecosphere, 12, e03297. 10.1002/ecs2.3297

[ece39648-bib-0070] Roffler, G. H. , & Gregovich, D. P. (2018). Wolf space use during denning season on Prince of Wales Island, Alaska. Wildlife Biology, 2018(1), wlb.00468. 10.2981/wlb.00468

[ece39648-bib-0071] Roffler, G. H. , Gregovich, D. P. , & Larson, K. R. (2018). Resource selection by coastal wolves reveals the seasonal importance of seral forest and suitable prey habitat. Forest Ecology and Management, 409, 190–201. 10.1016/j.foreco.2017.11.025

[ece39648-bib-0072] Roffler, G. H. , Waite, J. N. , Pilgrim, K. L. , Zarn, K. E. , & Schwartz, M. K. (2019). Estimating abundance of a cryptic social carnivore using spatially explicit capture–recapture. Wildlife Society Bulletin, 43(1), 31–41. 10.1002/wsb.953

[ece39648-bib-0073] Ruprecht, J. S. , Ausband, D. E. , Mitchell, M. S. , Garton, E. O. , & Zager, P. (2012). Homesite attendance based on sex, breeding status, and number of helpers in gray wolf packs. Journal of Mammalogy, 93(4), 1001–1005. 10.1644/11-MAMM-A-330.1

[ece39648-bib-0074] Schoen, J. , & Kirchhoff, M. (2016). Sitka black‐tailed deer. In M. Smith (Ed.), The ecological atlas of Southeast Alaska (pp. 151–153). Audubon Alaska.

[ece39648-bib-0075] Seal, U. S. , Mech, L. D. , & Van Ballenberghe, V. (1975). Blood analyses of wolf pups and their ecological and metabolic interpretation. Journal of Mammalogy, 56(1), 64–75. 10.2307/1379606 1113052

[ece39648-bib-0076] Shanley, C. S. , Eacker, D. R. , Reynolds, C. P. , Bennetsen, B. M. B. , & Gilbert, S. L. (2021). Using LiDAR and Random Forest to improve deer habitat models in a managed forest landscape. Forest Ecology and Management, 499, 119580. 10.1016/j.foreco.2021.119580

[ece39648-bib-0077] Shannon, C. E. (1948). A mathematical theory of communication. The Bell System Technical Journal, 27, 379–423.

[ece39648-bib-0078] Shibuya, H. , Collins, B. K. , Huang, T. H. M. , & Johnson, G. S. (1994). A polymorphic (AGGAAT)n tandem repeat in an intron of the canine von Willebrand factor gene. Animal Genetics, 25, 122. 10.1111/j.1365-2052.1994.tb00094.x 8010530

[ece39648-bib-0079] Sidorovich, V. , Schnitzler, A. , Schnitzler, C. , Rotenko, I. , & Holikava, Y. (2017). Responses of wolf feeding habits after adverse climatic events in central‐western Belarus. Mammalian Biology, 83, 44–50. 10.1016/j.mambio.2016.11.012

[ece39648-bib-0080] Spaulding, R. L. , Krausman, P. R. , & Ballard, W. B. (1998). Summer diet of Gray Wolves, *Canis lupus*, in northwestern Alaska. Canadian Field‐Naturalist, 112(2), 262–266.

[ece39648-bib-0081] Stahler, D. R. , & Smith, D. W. (2006). Foraging and feeding ecology of the gray wolf (*Canis lupus*): Lessons from Yellowstone National Park, Wyoming, USA. The Journal of Nutrition, 136(7 suppl), 1923S–1926S. 16772460 10.1093/jn/136.7.1923S

[ece39648-bib-0082] Steenweg, R. , Gillingham, M. P. , Parker, K. L. , & Heard, D. C. (2015). Considering sampling approaches when determining carnivore diets: the importance of where, how, and when scats are collected. Mammal Research, 60(3), 207–216. 10.1007/s13364-015-0222-4

[ece39648-bib-0083] Suring, L. H. , Degayner, E. J. , Flynn, R. W. , Kirchhoff, M. D. , Schoen, J. W. , & Shea, L. C. (1992). Habitat capability model for Sitka black‐tailed deer in Southeast Alaska: Winter habitat . Version 6.5 April 1992. US Forest Service, Region 10, Juneau, AK. http://www.fs.usda.gov/r10

[ece39648-bib-0084] Szepanski, M. M. , Ben‐David, M. , & Van Ballenberghe, V. (1999). Assessment of anadromous salmon resources in the diet of the Alexander Archipelago wolf using stable isotope analysis. Oecologia, 120(3), 327–335. 10.1007/s004420050866 28308010

[ece39648-bib-0085] Theberge, J. B. , & Cottrell, T. J. (1973). Food habits of wolves in Kluane National park. Arctic, 30(3), 189–191.

[ece39648-bib-0086] Theuerkauf, J. (2009). What drives wolves: Fear or hunger? Humans, diet, climate and wolf activity patterns. Ethology, 115(7), 649–657. 10.1111/j.1439-0310.2009.01653.x

[ece39648-bib-0087] U.S. Fish and Wildlife Service . (2020). Endangered and threatened wildlife and plants; 90‐day findings for three species. Federal Register, 141(86), 40186–40187.

[ece39648-bib-0088] Van Ballenberghe, V. , & Mech, L. D. (1975). Weights, growth, and survival of timber wolf pups in Minnesota. Journal of Mammalogy, 56(1), 44–63. 10.2307/1379605 1113051

[ece39648-bib-0089] Van Oosterhout, C. , Hutchinson, W. F. , Wills, D. P. M. , & Shipley, P. (2004). MICRO‐CHECKER: Software for identifying and correcting genotyping errors in microsatellite data. Molecular Ecology Notes, 4(3), 535–538. 10.1111/j.1471-8286.2004.00684.x

[ece39648-bib-0090] Weaver, J. J. , & Fritts, S. H. (1979). Comparison of coyote and wolf scat diameters. The Journal of Wildlife Management, 43(3), 786–788.

[ece39648-bib-0091] Wictum, E. , Kun, T. , Lindquist, C. , Malvick, J. , Vankan, D. , & Sacks, B. (2013). Developmental validation of DogFiler, a novel multiplex for canine DNA profiling in forensic casework. Forensic Science International: Genetics, 7(1), 82–91. 10.1016/j.fsigen.2012.07.001 22832398

[ece39648-bib-0092] Zimmermann, B. , Nelson, L. , Wabakken, P. , Sand, H. , & Liberg, O. (2014). Behavioral responses of wolves to roads: Scale‐dependent ambivalence. Behavioral Ecology, 25(6), 1353–1364. 10.1093/beheco/aru134 25419085 PMC4235582

