# Supplementary material for: Variation in adult and pup wolf diets at natal den sites is influenced by forest composition and configuration
Source: Ecol Evol. 2023 Jan 11;13(1):e9648. doi: 10.1002/ece3.9648 (PMC9834010; doi:10.1002/ece3.9648)

**Supporting Information**

**TABLE S1.** Wolf diet composition from metabarcoding of fecal DNA (identified to most specific taxonomic category), and groupings for analyses, Prince of Wales Island, Alaska 2014–2020. Proportions are displayed are the occurrence per item (O/I) index, the occurrence per feces (O/F) index, and the relative read abundance (RRA) estimates with the number (N) of occurrences per diet item.

| Diet item grouping | Diet items | Common name | N | O/I | O/F | RRA |
| --- | --- | --- | --- | --- | --- | --- |
| Amphibian | *Anaxyrus boreas* | Western toad | 1 | 0.129 | 0.186 | 0.002 |
| Bear | *Ursus americanus* | black bear | 56 | 7.235 | 10.409 | 2.469 |
| Beaver | *Castor canadensis* | beaver | 122 | 15.762 | 22.677 | 11.202 |
| Bird | *Anas* sp. | dabbling duck | 1 | 0.129 | 0.186 | 0.002 |
|  | *Antigone canadensis* | sandhill crane | 2 | 0.258 | 0.372 | 0.003 |
|  | *Ardea herodias* | blue heron | 1 | 0.129 | 0.186 | 0.084 |
|  | *Chen caerulescens caerulescens* | lesser snow goose | 3 | 0.388 | 0.558 | 0.257 |
|  | *Denragapus fuliginosus* | sooty grouse | 1 | 0.129 | 0.186 | 0.058 |
|  | *Falcipennis canadensis franklinii* | spruce grouse | 1 | 0.129 | 0.186 | 0.044 |
|  | *Haliaeetus leucocephalus* | bald eagle | 16 | 2.067 | 2.974 | 0.726 |
|  | *Junco hyemalis* | dark-eyed junco | 5 | 0.646 | 0.929 | 0.064 |
|  | *Melospiza melodia* | song sparrow | 1 | 0.129 | 0.186 | 0.005 |
|  | *Passeriformes* sp. | passerine | 1 | 0.129 | 0.186 | 0.006 |
|  | *Pica* sp*.* | magpie | 1 | 0.129 | 0.186 | 0.009 |
|  | *Sturnus vulgaris* | common starling | 1 | 0.129 | 0.186 | 0.005 |
|  | *Turdus migratorius* | thrush | 1 | 0.129 | 0.186 | 0.001 |
| Deer | *Odocoileus hemionus* | Sitka black-tailed deer | 468 | 60.465 | 86.989 | 76.352 |
| Domestic | *Gallus gallus* | chicken | 2 | 0.258 | 0.372 | 0.263 |
|  | *Sus scrofa domesticus* | pig | 3 | 0.388 | 0.558 | 0.130 |
| Marine mammal | *Enhydra lutris* | sea otter | 6 | 0.775 | 1.115 | 0.997 |
|  | *Eumetopias jubatus* | Steller sea lion | 1 | 0.129 | 0.186 | 0.186 |
|  | *Phoca vitulina* | harbor seal | 8 | 1.034 | 1.487 | 0.867 |
| Microtine | *Microtus* sp. | microtus sp. | 9 | 1.163 | 1.673 | 0.093 |
|  | *Peromyscus keeni* | Northwestern deer mouse | 3 | 0.388 | 0.558 | 0.052 |
| Mustelid | *Lontra canadensis* | river otter | 21 | 2.713 | 3.903 | 2.561 |
|  | *Martes americana* | American marten | 2 | 0.258 | 0.372 | 0.146 |
|  | *Neovison vison* | American mink | 9 | 1.163 | 1.673 | 1.325 |
| Other fish | *Cottidae* sp*.* | sculpin | 3 | 0.388 | 0.558 | 0.558 |
|  | *Xeneretmus latifrons* | blacktip poacher | 1 | 0.129 | 0.186 | 0.186 |
| Salmon | *Oncorhynchus gorbuscha* | pink salmon | 13 | 1.680 | 2.416 | 0.926 |
|  | *Oncorhynchus kisutch* | coho salmon | 7 | 0.904 | 1.301 | 0.177 |
|  | *Oncorhynchus mykiss* | rainbow trout | 1 | 0.129 | 0.186 | 0.055 |
|  | *Oncorhynchus* sp. | salmon | 2 | 0.258 | 0.372 | 0.178 |

**TABLE S2.** Landcover composition (proportion area), road density (km/km^2^), and area (km^2^) of wolf denning season home ranges (April 15–July 31), Prince of Wales Island, Alaska, 2015–2018. High-volume old-growth forest (HVPOG), medium-volume old-growth forest (MVPOG), low-volume old-growth forest (LVPOG), Non-forest vegetation (NF VEG), young-growth less than 25 years old (YG ≤25), young-growth more than 25 years old (YG >25), non-vegetated areas (NONVEG), closed roads (CLOSED), roads open to high clearance vehicles (HC ROADS), roads open to passenger vehicles (PASS ROADS).

| Year | Pack | Den ID | km^2^ | HVPOG | MVPOG | LVPOG | NF VEG | YG≤25 | YG>25 | NONVEG | CLOSED | HC ROADS | PASS ROADS |
| --- | --- | --- | --- | --- | --- | --- | --- | --- | --- | --- | --- | --- | --- |
| 2015 | Honker | 2 | 190 | 0.19 | 0.15 | 0.14 | 0.33 | 0.04 | 0.09 | 0.07 | 0.27 | 0.22 | 0.07 |
| 2016 | Hydaburg | 8 | 220 | 0.12 | 0.15 | 0.08 | 0.24 | 0.11 | 0.23 | 0.07 | 1.00 | 0.01 | 0.07 |
| 2016 | Ratz | 6 | 251 | 0.23 | 0.11 | 0.04 | 0.26 | 0.08 | 0.22 | 0.06 | 0.38 | 0.39 | 0.22 |
| 2016 | Sandy Beach | 5 | 60 | 0.18 | 0.15 | 0.05 | 0.16 | 0.06 | 0.39 | 0.01 | 0.73 | 0.52 | 0.20 |
| 2016 | Snow Pass | 9 | 5 | 0.02 | 0.22 | 0.30 | 0.32 | 0.00 | 0.00 | 0.13 | 0.00 | 0.00 | 0.00 |
| 2016 | Staney | 7 | 175 | 0.25 | 0.12 | 0.06 | 0.16 | 0.06 | 0.30 | 0.04 | 0.70 | 0.24 | 0.25 |
| 2017 | Hydaburg | 8 | 220 | 0.12 | 0.15 | 0.08 | 0.24 | 0.11 | 0.23 | 0.07 | 1.00 | 0.01 | 0.07 |
| 2017 | Old Frank's | 11 | 139 | 0.18 | 0.13 | 0.06 | 0.37 | 0.16 | 0.04 | 0.07 | 0.47 | 0.14 | 0.00 |
| 2017 | Sandy Beach | 10 | 101 | 0.12 | 0.15 | 0.06 | 0.17 | 0.07 | 0.36 | 0.07 | 0.74 | 0.40 | 0.28 |
| 2017 | Snow Pass | 9 | 5 | 0.02 | 0.22 | 0.30 | 0.32 | 0.00 | 0.00 | 0.13 | 0.00 | 0.00 | 0.00 |
| 2017 | Trocadero | 12 | 28 | 0.15 | 0.21 | 0.06 | 0.30 | 0.09 | 0.04 | 0.14 | 0.82 | 0.00 | 0.00 |
| 2018 | Honker | 2 | 190 | 0.19 | 0.15 | 0.14 | 0.33 | 0.04 | 0.09 | 0.07 | 0.27 | 0.22 | 0.07 |
| 2018 | Hydaburg | 8 | 220 | 0.12 | 0.15 | 0.08 | 0.24 | 0.11 | 0.23 | 0.07 | 1.00 | 0.01 | 0.07 |
| 2018 | Old Frank's | 11 | 139 | 0.18 | 0.13 | 0.06 | 0.37 | 0.16 | 0.04 | 0.07 | 0.47 | 0.14 | 0.00 |
| 2018 | Staney | 3 | 53 | 0.16 | 0.10 | 0.10 | 0.19 | 0.09 | 0.27 | 0.10 | 0.68 | 0.25 | 0.47 |

**TABLE S3.** Landcover composition (proportion area), road density (km/km^2^), and area (km^2^) of wolf annual home ranges, Prince of Wales Island, Alaska, 2015–2018. High-volume old-growth forest (HVPOG), medium-volume old-growth forest (MVPOG), low-volume old-growth forest (LVPOG), Non-forest vegetation (NF VEG), young-growth less than 25 years old (YG ≤25), young-growth more than 25 years old (YG >25), non-vegetated areas (NONVEG), closed roads (CLOSED), roads open to high clearance vehicles (HC ROADS), roads open to passenger vehicles (PASS ROADS).

| Year | Pack | Den ID | km^2^ | HVPOG | MVPOG | LVPOG | NF VEG | YG≤25 | YG>25 | NONVEG | CLOSED | HC ROADS | PASS ROADS |
| --- | --- | --- | --- | --- | --- | --- | --- | --- | --- | --- | --- | --- | --- |
| 2015 | Honker | 2 | 671 | 0.23 | 0.13 | 0.08 | 0.28 | 0.06 | 0.14 | 0.08 | 0.31 | 0.30 | 0.19 |
| 2016 | Hydaburg | 8 | 185 | 0.12 | 0.15 | 0.08 | 0.24 | 0.11 | 0.23 | 0.07 | 0.85 | 0.00 | 0.07 |
| 2016 | Ratz | 6 | 405 | 0.21 | 0.12 | 0.06 | 0.27 | 0.07 | 0.20 | 0.06 | 0.35 | 0.39 | 0.21 |
| 2016 | Sandy Beach | 5 | 179 | 0.18 | 0.14 | 0.06 | 0.22 | 0.08 | 0.28 | 0.04 | 0.60 | 0.44 | 0.19 |
| 2016 | Snow Pass | 9 | 38 | 0.09 | 0.22 | 0.14 | 0.18 | 0.00 | 0.31 | 0.07 | 0.26 | 0.00 | 0.00 |
| 2016 | Staney | 7 | 205 | 0.16 | 0.10 | 0.10 | 0.15 | 0.06 | 0.37 | 0.07 | 0.68 | 0.47 | 0.25 |
| 2017 | Hydaburg | 8 | 185 | 0.12 | 0.15 | 0.08 | 0.24 | 0.11 | 0.23 | 0.07 | 0.85 | 0.00 | 0.07 |
| 2017 | Old Frank's | 11 | 139 | 0.18 | 0.13 | 0.06 | 0.37 | 0.16 | 0.04 | 0.07 | 0.47 | 0.14 | 0.00 |
| 2017 | Sandy Beach | 10 | 179 | 0.18 | 0.14 | 0.06 | 0.22 | 0.08 | 0.28 | 0.04 | 0.60 | 0.44 | 0.19 |
| 2017 | Snow Pass | 9 | 38 | 0.09 | 0.22 | 0.14 | 0.18 | 0.00 | 0.31 | 0.07 | 0.26 | 0.00 | 0.00 |
| 2017 | Trocadero | 12 | 688 | 0.13 | 0.12 | 0.07 | 0.37 | 0.06 | 0.13 | 0.12 | 0.50 | 0.06 | 0.14 |
| 2018 | Honker | 2 | 671 | 0.23 | 0.13 | 0.08 | 0.28 | 0.06 | 0.14 | 0.08 | 0.31 | 0.30 | 0.19 |
| 2018 | Hydaburg | 8 | 185 | 0.12 | 0.15 | 0.08 | 0.24 | 0.11 | 0.23 | 0.07 | 0.85 | 0.00 | 0.07 |
| 2018 | Old Frank's | 11 | 139 | 0.18 | 0.13 | 0.06 | 0.37 | 0.16 | 0.04 | 0.07 | 0.47 | 0.14 | 0.00 |
| 2018 | Staney | 3 | 205 | 0.16 | 0.10 | 0.10 | 0.15 | 0.06 | 0.37 | 0.07 | 0.68 | 0.47 | 0.25 |

Figure S1. Individual-based rarefaction curves of species diversity (H’) of wolf diet items as a function of sample size for each pack; Southeast Alaska, USA, 2015–2018.


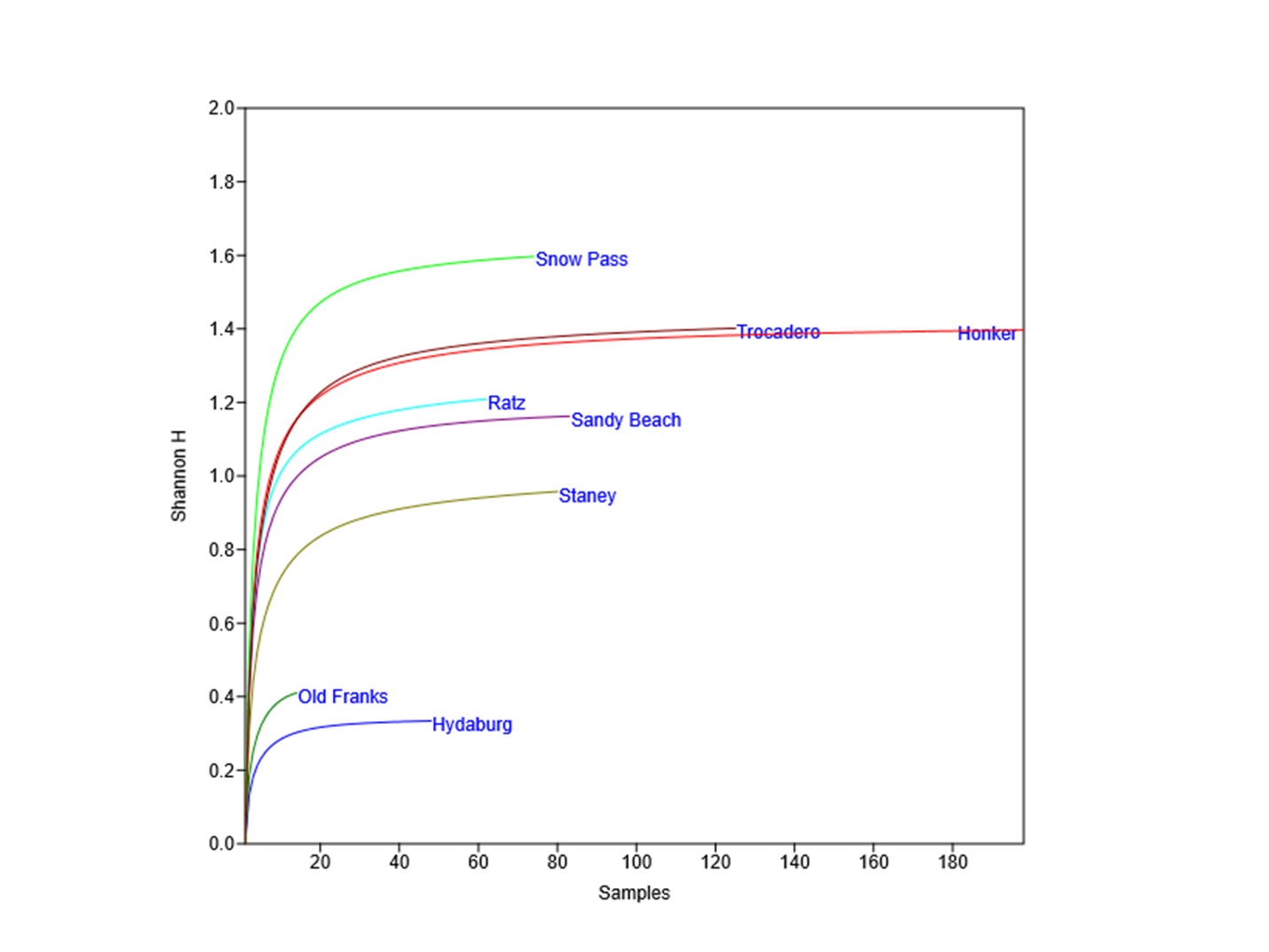


Figure S2. Diet composition by wolf pack based on the relative frequency (occurrence per item (O/I) index) of diet items identified in pup and adult wolf scats collected at active dens during the denning season (April 15–July 31), Prince of Wales Island, Alaska, 2015–2018.


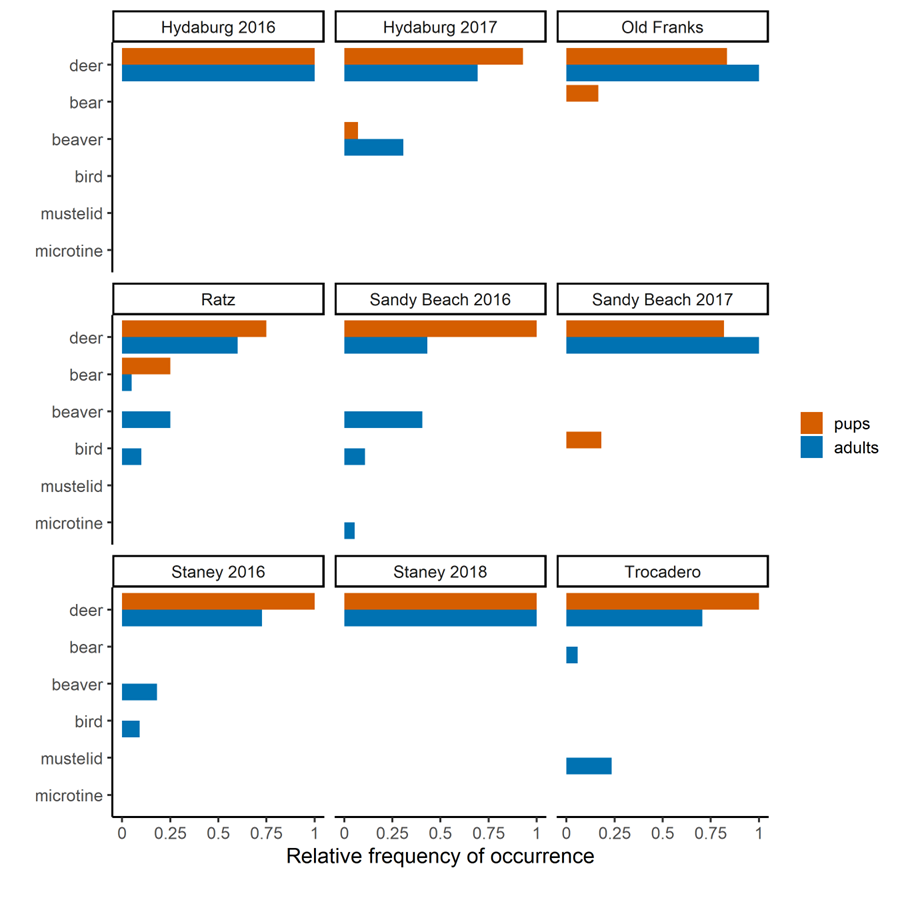

Supplement: Supplementary file 1 — AppendixS1 [file ECE3-13-e9648-s001.docx]
